# A genome-skimmed phylogeny of a widespread bryozoan family, Adeonidae

**DOI:** 10.1186/s12862-019-1563-4

**Published:** 2019-12-27

**Authors:** Russell J. S. Orr, Marianne N. Haugen, Björn Berning, Philip Bock, Robyn L. Cumming, Wayne K. Florence, Masato Hirose, Emanuela Di Martino, Mali H. Ramsfjell, Maja M. Sannum, Abigail M. Smith, Leandro M. Vieira, Andrea Waeschenbach, Lee Hsiang Liow

**Affiliations:** 1 Natural History Museum, University of Oslo, Oslo, Norway; 2 Centre for Ecological and Evolutionary Synthesis, Department of Biosciences, University of Oslo, Oslo, Norway; 3 Geoscience Collections, Upper Austrian State Museum, Linz, Austria; 4 Museum Victoria, Melbourne, Victoria Australia; 5 Museum of Tropical Queensland, Townsville, Australia; 6 Department of Research and Exhibitions, Iziko Museums of South Africa, Cape Town, South Africa; 7 School of Marine Biosciences, Kitasato University, Kanagawa, Japan; 8 Department of Marine Science, University of Otago, Dunedin, New Zealand; 9 Department of Zoology, Universidade Federal de Pernambuco, Recife, Brazil; 10 Department of Life Sciences, Natural History Museum, London, UK

**Keywords:** Cheilostome bryozoans, Genome skimming, Mitogenome, Phylogeny, rRNA

## Abstract

**Background:**

Understanding the phylogenetic relationships among species is one of the main goals of systematic biology. Simultaneously, credible phylogenetic hypotheses are often the first requirement for unveiling the evolutionary history of traits and for modelling macroevolutionary processes. However, many non-model taxa have not yet been sequenced to an extent such that statistically well-supported molecular phylogenies can be constructed for these purposes. Here, we use a genome-skimming approach to extract sequence information for 15 mitochondrial and 2 ribosomal operon genes from the cheilostome bryozoan family, Adeonidae, Busk, 1884, whose current systematics is based purely on morphological traits. The members of the Adeonidae are, like all cheilostome bryozoans, benthic, colonial, marine organisms. Adeonids are also geographically widely-distributed, often locally common, and are sometimes important habitat-builders.

**Results:**

We successfully genome-skimmed 35 adeonid colonies representing 6 genera (*Adeona, Adeonellopsis, Bracebridgia, Adeonella, Laminopora* and *Cucullipora*). We also contributed 16 new, circularised mitochondrial genomes to the eight previously published for cheilostome bryozoans. Using the aforementioned mitochondrial and ribosomal genes, we inferred the relationships among these 35 samples. Contrary to some previous suggestions, the Adeonidae is a robustly supported monophyletic clade. However, the genera *Adeonella* and *Laminopora* are in need of revision: *Adeonella* is polyphyletic and *Laminopora* paraphyletically forms a clade with some *Adeonella* species. Additionally, we assign a sequence clustering identity using *cox1* barcoding region of 99% at the species and 83% at the genus level.

**Conclusions:**

We provide sequence data, obtained via genome-skimming, that greatly increases the resolution of the phylogenetic relationships within the adeonids. We present a highly-supported topology based on 17 genes and substantially increase availability of circularised cheilostome mitochondrial genomes, and highlight how we can extend our pipeline to other bryozoans.

## Background

A robust, well-supported species-level phylogeny is often one of the first key steps in understanding evolution above the level of a population. Many understudied taxa have great potential for answering broad evolutionary questions, yet they frequently lack modern phylogenetic treatments. Some of these understudied taxa could also benefit from having their evolutionary relationships better resolved in order to inform ecological questions or even management decisions. The phylum Bryozoa is one such taxon. It has been used to study key evolutionary questions [1–3], but as a whole, it is still far behind other comparable taxa in terms of available robust phylogenetic hypotheses estimated using molecular data. As has been shown for other taxa such as damselflies [4], molluscs [5] and fish [6], it is clear that the phylogenetic hypotheses for bryozoans built upon only morphological information are often incongruent with those based on independent molecular data [7–9]. This is because any subset of characters reflects only part of the evolutionary history of the groups in question. There are also strong indications that the integration of different kinds of data gives more information and better estimates of uncertainty of patterns of diversification [10, 11]. Hence, there is a general move towards the integration of morphological, temporal (including fossil) and molecular data in our common quest for understanding phylogenetic relationships including the timing and rates of evolutionary change [12, 13]. Here, we begin to alleviate the lack of robust phylogenetic hypotheses among bryozoans by focusing our efforts on an ecologically conspicuous family, the Adeonidae.

Adeonidae is a relatively species-rich family whose fossil record is known from Cenozoic seas since the Ypresian, 56.0–47.8 million years ago [14]. Members of this tropical to subtropical family are widespread and often locally abundant. They create habitats that support diverse communities [15], and contribute significantly to shelf carbonate dynamics [16]. This family is currently divided into 10 extant genera with 106 described extant species [17]. Previously, the Adeonidae were separated into two distinct families by Gregory (1893): Adeonidae (including *Adeona, Adeonellopsis, Bracebridgia, Cucullipora* and *Reptadeonella*) and Adeonellidae, Gregory 1893 (comprising the genera *Adeonella* and *Laminopora*). This separation was corroborated by Cook (1973) who showed that zooids in the Adeonidae exhibit an umbonulomorph development, i.e. zooids in which the polypide and an exposed frontal membrane form during early ontogeny, over which an umbonuloid frontal shield develops at a later stage. In contrast, in the Adeonellidae the zooids show a lepraliomorph development, in which an internal lepralioid frontal shield forms prior to the development of the polypide and ascus. These differences in zooid development and frontal shield formation have historically been considered one of the most important characters in high-level cheilostome systematics (e.g. [18, 19]), and it was commonly assumed that genera belonging to the same family have the same frontal shield state. However, both types of zooidal development and frontal shields may exist in closely related taxa [19]. For instance, in the genus *Parkermavella* some species exhibit entirely lepralioid frontal shields while others show mixed types, i.e. partly lepralioid shields with varying extents of umbonuloid areas [20]. These findings dispute the usefulness and stability of this trait for taxonomic classification at a level higher than that of the species (see e.g. [21]). However, testing the monophyly of Adeonidae *s.l.* using characters independent of morphological traits, i.e. genetic molecular studies, have not been conducted to date. The same applies to the bryozoan order Cheilostomata as a whole: though previous phylogenetic analyses have indicated that the frontal shield may have its limitations as an evolutionary trait that is stable enough to be used in family systematics [7, 22], this is yet to be corroborated using a highly-supported tree topology.

Our main aim here is to estimate the phylogenetic relationships among adeonid bryozoans (i.e. Adeonidae *s.s.* and Adeonellidae *s.s.*), utilising shot-gun sequencing techniques that have become commonplace among other taxa [23–25]. More recent efforts in building molecular phylogenies of bryozoans, in particular the largest group of extant bryozoans, the cheilostomes, have at most used 7 genes [7, 26–28], using PCR-based methods. Preserved bryozoan material often contains fragmented DNA of the target taxon, making full length amplification problematic. Further, low sequence conservation among bryozoan species impedes the use of “universal primers” for PCR [7, 28].

To alleviate the deficiency of non-morphological data for resolving relationships among species of the Adeonidae *s.l.,* we genome-skimmed (i.e. low coverage genomic shotgun sequencing of a target taxon to recover DNA sequences from high-copy genes) 35 adeonid colonies representing seven named genera (five from Adeonidae *s.s.* and two from Adeonellidae *s.s.*) and targeted genes from the mitochondria and (nuclear) ribosomal operon, then built a phylogeny based on these. We discuss to what extent systematics based on adeonid morphology is reflected by their molecular phylogeny and how the latter changes our view of the evolution of this widespread family.

## Results

**Fig. 1:**
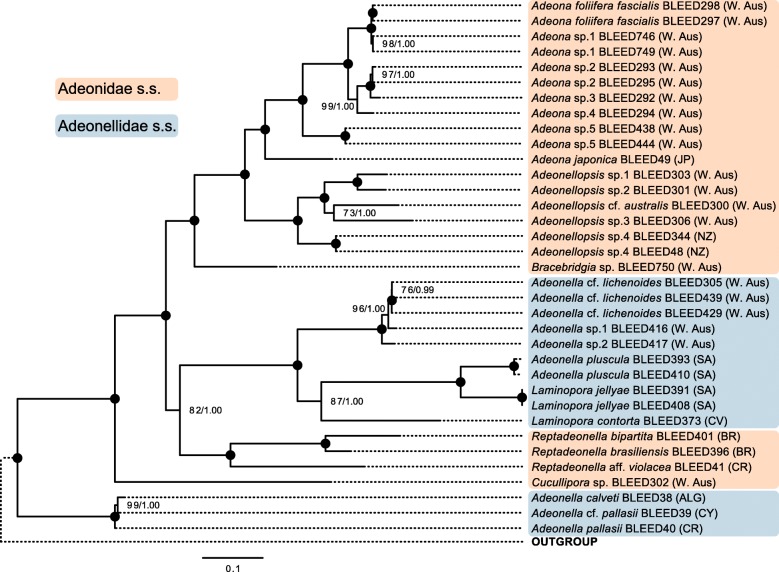
The phylogeny of adeonids based on 17 genes. Maximum likelihood topology of 35 adeonid ingroup taxa with 10,531 nucleotide and amino acid characters inferred using RAxML. The numbers on the internal nodes are ML bootstrap values (BP from RAxML) followed by posterior probabilities (PP from MrBayes). Black circles indicate 100BP and 1.00PP. The placement and statistical support for the outgroup is taken from Additional file 1: Figure S3 and represented with a stippled line. Adeonellidae also represents the lepralioid frontal shield form as does Adeonidae the umbonuloid form (note the frontal shield form has been inferred from samples other than those represented in the study and that the *Cucullipora* frontal shield form is unknown). Abbreviations for countries: W. Aus = Western Australia, JP = Japan, NZ = New Zealand, SA = South Africa, CV = Cape Verde, BR = Brazil, CR = Croatia, ALG = Algeria, and CY = Cyprus. The scale bare represents substitutions per site

We successfully sequenced 35 colonies belonging to the Adeonidae, assembled and annotated the majority of the mitochondrial and two rRNA operon genes (Table 1 and Fig. 1). These data are deposited at NCBI with accession numbers. Sixteen of the mitochondrial genomes of the 35 colonies were circularized.

**Table 1:** Taxa used in this study

Taxon	BLEED nr.	Location	Accession nr.	Circularised mitogenome (bp)	Genes
*Adeona foliifera fascialis*	298	W. Aus	MK894382, MK894346, MN131169	14,577	17
*Adeona foliifera fascialis*	297	W. Aus	MK894378, MK894344, MN131148	14,574	16
*Adeona* sp.1	746	W. Aus	MK894379, MN131151	14,578	15
*Adeona* sp.1	749	W. Aus	MK894381, MK894345, MN131147	14,578	16
*Adeona* sp.2	293	W. Aus	MK894376, MK894342, MN131159	14,671	17
*Adeona* sp.2	295	W. Aus	MK894377, MK894343, MN131153	14,701	17
*Adeona* sp.3	292	W. Aus	MK894380, MK894375, MN131150	14,693	17
*Adeona* sp.4	294	W. Aus	MK894383, MK894374, MN131155	14,663	17
*Adeona* sp.5	438	W. Aus	MK894384, MK894347, MN131171	16,229	17
*Adeona* sp.5	444	W. Aus	MK894385, MK894348, MN131154		17
*Adeona japonica*	49	JP	MK894386, MK894372, MN131175		17
*Adeonellopsis* sp.1	303	W. Aus	MK894404, MK894364, MN131166		16
*Adeonellopsis* sp.2	301	W. Aus	MK894405, MK894365, MN131149		17
*Adeonellopsis* cf. *australis*	300	W. Aus	MK894402, MK894362, MN131167		17
*Adeonellopsis* sp.3	306	W. Aus	MK894403, MK894363, MN131172		13
*Adeonellopsis* sp.4	344	NZ	MK894406, MK894366, MN131174		17
*Adeonellopsis* sp.4	48	NZ	MK894407, MK894367, MN131152	15,198	17
*Bracebridgia* sp.	750	W. Aus	MK894390, MK894370, MN131163		14
*Adeonella* cf. *lichenoides*	305	W. Aus	MK894399, MK894355, MN131170		17
*Adeonella* cf. *lichenoides*	439	W. Aus	MK894396, MK894352, MN131162		17
*Adeonella* cf. *lichenoides*	429	W. Aus	MK894398, MK894353, MN131146		17
*Adeonella* sp.1	416	W. Aus	MK894397, MK894354, MN131144		17
*Adeonella* sp.2	417	W. Aus	MK894395, MK894351, MN131176		16
*Adeonella pluscula*	393	SA	MK894391, MK894349, MN131157	19,241	17
*Adeonella pluscula*	410	SA	MK894392, MK894350, MN131161	19,436	16
*Laminopora jellyae*	391	SA	MK894393, MK894357, MN131177–81		13
*Laminopora jellyae*	408	SA	MK894394, MK894358, MN131182–87		15
*Laminopora contorta*	373	CV	MK894400, MK894356, MN131158		17
*Reptadeonella bipartita*	401	BR	MK894388, MK894369, MN131173		15
*Reptadeonella brasiliensis*	396	BR	MK894389, MK894373, MN131164	16,957	16
*Reptadeonella* aff. *violacea*	41	CR	MK894387, MK894371, MN131160	14,696	17
*Cucullipora* sp.	302	W. Aus	MK894401, MK894368, MN131156	18,244	16
*Adeonella calveti*	38	ALG	MK894408, MK894359, MN131168	16,859	17
*Adeonella* cf. *pallasii*	39	CY	MK894409, MK894360, MN131145		17
*Adeonella pallasii*	40	CR	MK894410, MK894361, MN131165		16
*Bugula neritina*			NC_010197		15
*Celleporella hyalina*			JN680948, NC_018344		15
*Flustra foliacea*			FJ196110, NC_016722		16
*Flustrellidra hispida*			FJ409601, FJ409577, NC_008192		15
*Membranipora grandicella*			NC_018355		14
*Pectinatella magnifica*			FJ409600, FJ409576, NC_038192		16
*Tubulipora flabellaris*			EU650325, DQ333340, NC_015646		15
*Watersipora subtorquata*			JN680947, JN681042, NC_011820		16

In-group taxa generated for this study are shown in white, whilst outgroup taxa are shown in grey. BLEED stands for Bryozoan Lab for Ecology, Evolution and Development and BLEED numbers are numerical tags for the specimens. Accession nr. refer to those at NCBI. Abbreviations for countries (descending order): *W. Aus* Western Australia, *JP* Japan, *NZ* New Zealand, *SA* South Africa, *CV* Cape Verde, *BR* Brazil, *CR* Croatia, *ALG* Algeria, and *CY* Cyprus. The size, in base pairs (bp), of the mitogenome are only shown if it is closed/circularised. Genes, represents the number of genes, of maximum 17, recovered and used in the alignments for each taxon. For an expanded overview of metadata see Additional file 2: Table S1

We show the rRNA (Additional file 1: Figure S1) and mitogenome (Additional file 1: Figure S2) ingroup tree inferences to have more congruent topologies than expected by chance with an *I*_*cong*_ index of 2.89 and a *P*-value of 2.82. The result indicates a congruent evolution and supports the concatenation of the rRNA and mitogenome data. As such, we only present the results for the 17 gene datasets in the main text.

Our ingroup taxa form a fully supported monophyletic clade (100 bootstrap (BS) / 1.00 Posterior Probability (PP)), when the rRNA+mitogenome dataset with outgroup taxa is used for inference (Additional file 1: Figure S3; 43 taxa and 10,259 characters).

The clade consisting of colonies of *Adeonella calveti, Adeonella pallasii* and *A.* cf. *pallasii* from the Mediterranean are the first to diverge within Adeonidae (100BS/1.00PP; Fig. 1 based on 35 taxa and 10,531 characters). The first taxon to branch off from this basal clade is *Cucullipora*, excluded from the remaining adeonids with full support (100BS/1.00PP). The latter are divided into two monophyletic clades; The first clade with medium support (82BS/1.00PP) constitutes members of the genera *Adeonella*, *Laminopora*, and *Reptadeonella.* The fully-supported, second clade (100BS/1.00PP) comprises *Adeona*, *Adeonellopsis* and *Bracebridgia*.

In the first clade derived from *Cucullipora*, three species attributed to the genus *Reptadeonella* form a fully supported monophyly (100BS/1.00PP), which is sister to the fully-supported clade containing a mixture of *Adeonella* and *Laminopora* (100BS/1.00PP). Within the latter, the South African taxa form a fully-supported grouping (100BS/1.00PP) within a larger monophyly (87BS/1.00PP) encompassing *Laminopora contorta* from Cape Verde. Together with the Western Australian clade of *Adeonella* (100BS/1.00PP) this forms a larger monophyletic grouping (100BS/1.00PP).

In the second clade split from *Cucullipora*, *Bracebridgia* is inferred as the most basal taxon and excluded from the *Adeona* and *Adeonellopsis* monophyly with full support (100BS/1.00PP). The *Adeonellopsis* clade (100BS/1.00PP), constitutes species from New Zealand and Western Australia. The fully supported *Adeona* grouping (100BS/1.00PP) includes the Japanese species *Adeona japonica* (previously *Adeonellopsis japonica*; see discussion) placing basal to a clade of Western Australian species (100BS/1.00PP).

Clustering at varying levels of nucleotide identity for 18 s V4, 28 s D1-D2 and *cox1* (Additional file 1: Figure S4) show different levels of sequence conservation. The 18 s V4 region can recover adeonid morphogenera at 99% clustering identity; 28 s D1-D2 cannot replicate the adeonid morpho-taxonomical ranks at any clustering level; but *cox1* captures adeonid morphospecies similarities at the 99% clustering level and genera at 83% for our dataset.

## Discussion

The interrelationships of adeonids have been controversial throughout the history of bryozoology [29–31], where its constituents have been repeatedly separated and (re)combined in two families, the Adeonidae s.s. and the Adeonellidae. With our new sequence data, we confirm that Adeonidae *s.l.* is a monophyletic family, regardless of the frontal shield states of its constituent members. The last major comparative morphological study of both adeonid and adeonellid genera was carried out by Cook (1973). Cook concluded that the Adeonellidae (*Adeonella* and *Laminopora*) differs radically from the Adeonidae s.s. (*Adeona*, *Adeonellopsis*, *Reptadeonella* and *Bracebridgia*) in the mode of development of the calcified frontal wall of the autozooid, in the heterochronic development of the polypide and orifice relative to the formation of the frontal shield, and in the homology of the frontal pore complex. However, Cook (1973) also anticipated that, with further studies on adeonids and other cheilostome taxa, the ontogeny of the frontal shield and protrusion apparatus may prove to be phylogenetically uninformative. Comparing the skeletal morphogenesis in the representatives (*Adeonella* and *Adeonellopsis)* of the two groups, Lidgard (1996) suggested that their similarities are greater than their differences. We observe that the genera assigned to the two families (the lepraliomorph Adeonellidae and the umbonulomorph Adeonidae s.s.) are distributed across our phylogeny. The *Adeonella calveti/pallasii* group with its purportedly lepralioid frontal shield forms a basal clade among the taxa we sequenced. The more derived *Adeonella* clade (which includes the lepraliomorph *Laminopora*) forms a monophyly with *Reptadeonella* (umbonulomorph). The clade constituting *Bracebridgia, Adeonellopsis* and *Adeona* are also umbonulomorph. With the presumably lepraliomorph group of European *Adeonella* at the base of the tree, the topology seems to suggest that lepraliomorph development is ancestral, and that umbonulomorph adeonids have evolved at least twice (1: *Bracebridgia, Adeonellopsis*, *Adeona*; 2: *Reptadeonella*; the type of frontal shield development in *Cucullipora* is unknown at present).

However, the progressive reduction of the umbonuloid component in lepralioid frontal shields over geological time, as well as studies on zooid and frontal shield ontogeny, suggest that lepraliomorph development may be the derived state (e.g. [19, 31]). Given our relatively limited sampling of the adeonids here, it is plausible we have not sampled the most basal extant taxa, which may be umbonulomorph. However, as extant adeonid taxa exhibit derived features (such as endozooidal brooding and extraembryonic nutrition, cf. [32]), and as the genus *Adeonella* presumably originated already in the Paleogene, it is likely that all potential ancestral lineages are extinct.

We would like to highlight, however, that the above assignments of the umbonulomorph or lepraliomorph states of development to the genera we have sequenced are based on the published literature [21, 31] and not the specimens or even the species depicted in our tree. We here abstain from over-interpreting the evolution of frontal shields on our current tree topology for the Adeonidae as further detailed studies on zooid ontogeny and frontal shield formation, particularly of the basal European clade of *Adeonella*, are necessary for a deeper understanding of the group.

We provide molecular evidence that at least the *Adeona* species (note that all our samples are from Western Australia) form a statistically supported clade which can be distinguished by their rounded, single-pore spiramen. Its sister clade, *Adeonellopsis,* which is represented by samples from both Australia and New Zealand, have distinct multiporous spiramina with marginal denticles. Interestingly, what has been called *Adeonellopsis japonica* has traits intermediate between *Adeona* and *Adeonellopsis,* where it appears that only gonozooids have a multiporous spiramen with marginal denticles, but not autozooids. Based on our phylogenetic inference (Fig. 1), *Adeonellopsis japonica* has a closer genetic affinity to the *Adeona* group and is better placed in that genus, given the intermediate nature of *A. japonica*’s key morphological traits.

Several morphogenera for which we have multiple species represented (*Adeona*, *Reptadeonella, Adeonellopsis)* have stood the test of independent molecular data. However, *Adeonella* did not stand up to the molecular scrutiny. In our statistically highly-supported tree (Fig. 1), *Laminopora* and the derived *Adeonella* could be considered a single genus (Additional file 1: Figure S4).

Based on limited available morphological traits and our molecular result, we suggest future studies consider synonymising *Laminopora* with the derived *Adeonella* group*.* Note, however, that the Mediterranean species attributed to *Adeonella* are at the base of our tree, dissected by *Cucullipora*, indicating *Adeonella* as polyphyletic. At this point, we do not have any morphological characters for distinguishing these basal *Adeonella* from those that are sister to *Reptadeonella.* The contradiction between morphotype and genotype for the two *Adeonella* clades demands a taxonomic revision of species attributed to this genus. Until distinguishing traits can be identified with further work, we suggest using molecular markers for assigning extant taxa with *Adeonella-*like morphology to either one of the branches. Further, assigning fossil samples to an *Adeonella* morphotype should be performed with caution. A substantial amount of work on both the basal group of ‘*Adeonella*’ (including *A. pallasii, A.* cf. *pallasii* and *A. calveti)* and the derived group (including *A.* cf. *lichenoides* and *Adeonella* sp. 1 and *Adeonella* sp. 2) is required to detail any distinguishing morphological differences that can be used to delimit these two clades. Given that the basal *Adeonella* clade contains the type species, the derived clade would either need a new name or formal synonymizing with *Laminopora*, though the taxonomic work necessary for this is beyond the scope of this current paper. However, the Adeonidae could serve as an example where we might cautiously conclude that morphogenera are, on average, good lineages, although exceptions can occur.

We have used several diverse outgroups to root our adeonid tree (Additional file 1: Figure S3), including a phylactolaemate (*Pectinatella*), a cyclostome (*Tubulipora),* a ctenostome (*Flustrellidra*) and also cheilostomes that are thought to be basal (*Membranipora*) and more derived with respect to adeonids (*Watersipora*). However, without wider taxon sampling it is only possible to speculate, based on morphological traits, what the living sister groups to the adeonids might be. In addition to Adeonidae, there is only one other extant family in the superfamily Adeonoidea, the Inversiulidae [33], which would be a natural sister group candidate. Some taxa attributed to Arachnopusiidae also have traits that are reminiscent of Adeonidae, including *Poricella,* some species of which have adeonid-like spiramina. Some taxa currently placed in the Exechonellidae (e.g. *Anarthropora*) have occasionally been placed in the Adeonidae [34]. *Metrarabdotos* (Metrarabdotosidae) and *Escharoides* (Exochellidae) have likewise been regarded as distant relatives of the Adeonidae [35]. Future studies aiming to further resolve the phylogenetic relationships within the Adeonidae should naturally include the more rarely occurring adeonid genera *Triporula, Kubaninella, Anarthropora* and *Dimorphocella*, and should also be more inclusive in the geographic sampling of the genera that we present here.

Clustering three typically used molecular barcoding regions at varying levels of sequence identity allows us to suggest an identity cut-off at the species and genus level, at least for the adeonids we sequenced. Our results suggest that we can use a 99% clustering identity using the 18 s V4 region and/or 83% using *cox1* to delimit adeonid morphogenera; and a 99% clustering identity using *cox1* to delimit adeonid morphospecies. This differs from the somewhat arbitrary ‘universal’ clustering level at 97% identity for species and 90% for genus (based on 18 s V4), and reinforces that thresholds need to be determined separately for all groups to avoid either the under- or overestimation of species and genus diversity [36]. Interestingly, if we consider only genetic sequence data, *Adeonella pallasii*, *A.* cf. *pallasii* and *A. calveti* could be considered as a single species with diverse morphologies (see [37].). Short of carrying out crossing experiments to resolve the biological species problem [38], or common garden experiments such as those done by Jackson and Cheetham for a handful of cheilostomes [2], future studies should consider sequencing more genes from geographically and morphologically diverse representatives of this group of *Adeonella.* The task of increasing taxon- and gene-sampling naturally applies to other bryozoans as well.

Genome skimming is an effective approach for acquiring enough sequence information for building statistically robust phylogenetic hypotheses that are the bases for further investigations into evolutionary history and processes. Such an approach is relevant for understudied groups whose variation in morphological traits maybe less understood than those of more studied groups, such as mammals and plants. While easily observed morphological traits can give first insights into the systematics of a group, sequence data give many additional and independent characters that can help systematists refine their hypotheses. We show the value of genome-skimmed data for resolving the phylogeny of a cheilostome family for the first time. The ease of extracting whole mitochondrial genomes, for which seven, representing only five species, are currently published in NCBI, [39–44], is also promising.

## Conclusion

Significantly expanding taxon and gene sampling for bryozoans is within reach: we show here that genome-skimming greatly increased the resolution of the phylogenetic relationships within the adeonids, and can be easily extended to other bryozoans. We presented a highly-supported topology based on 17 genes, more than doubling previous best efforts to build bryozoan phylogenies in terms of sequence data [7, 28]. The ease of extracting whole mitochondrial genomes is also promising. As an added bonus, we also note that the taxa sequenced herein are now “barcoded” without using a standard barcoding protocol [45]. Hence, we suggest that Illumina sequencing can be used as an effective alternative to Sanger sequencing, for the barcoding of understudied taxa, such as bryozoans. However, molecular data is not a substitute for detailed taxonomic work and our study should be seen as complementary to taxonomic research. While this study is a small contribution to the genomic data of marine invertebrates, it could be considered a leap in the field of bryozoology.

## Methods and material

Tissue samples were fixed in 70–95% ethanol at the time of sampling to minimise DNA degradation. Sequenced material came mainly from Western Australia, with additional samples from Algeria, Brazil, Croatia, Cyprus, Japan, New Zealand and South Africa (Table 1). All samples were exported to Norway according to the protocols of the countries of origin and also adhere to the Nagoya Protocol. Each colony was subsampled for DNA isolation and separately for scanning electron microscopy (SEM), using a Hitachi TM4040PLus. We bleached the latter subsamples in diluted household bleach for a few hours to overnight to remove soft tissues, in order to document skeletal morphology. SEMs of dried samples were taken both pre- and post-bleaching (Additional file 3). All physical vouchers are deposited in a public collection at the Natural History Museum of Oslo, University of Oslo, and SEMs are available in the Online Supplementary Information (SI). Other metadata associated with our samples are reported in Additional file 2: Table S1.

### DNA isolation, sequencing, assembly and annotation

Ethanol-preserved samples were dried before genomic DNA was isolated using the DNeasy Blood and Tissue kit (QIAGEN, Germantown, MD, USA). Colonies were homogenised in lysis buffer, using a pestle, in the presence of proteinase-K. Genomic DNA were sequenced at the Norwegian Sequencing Centre (Oslo, Norway) using Illumina HiSeq4000 150 bp paired-end (PE) sequencing with a 350 bp insert size. Illumina HiSeq reads were quality- and adapter-trimmed using TrimGalore v0.4.4 (https://www.bioinformatics.babraham.ac.uk/projects/trim_galore/) and assembled with SPAdes 3.11.1 [46] with k-mers of 33, 55, 77, 99, and 121, before final genome polishing with Pilon [47]. The mitogenome and rRNA operon sequences for all 35 adeonids were identified with blastn in CLC main workbench 7 (Qiagen, Hilden, Germany). Eight bryozoan mitogenomes and 20 nuclear rRNA operons, previously deposited in NCBI were used as queries (Table 1, grey rows).

### Annotation and alignments

The 35 separate mitogenomes were annotated with Mitos2 [48]. There are two rRNA genes (rrnL and rrns) and 13 protein coding genes (*atp6, atp8, cox1, cox2, cox3, cob, nad1, nad2, nad3, nad4, nad4l, nad5,* and *nad6*). In addition, two rRNA operon genes (ssu (18 s) and lsu (28 s)) were identified and annotated using RNAmmer [49]. Hence, in total, 17 genes were used for phylogenetic inferences. The eight known bryozoan mitogenomes and rRNA operons (Table 1) were aligned with our 35 adeonids, constituting the out- and in-group taxa respectively. MAFFT [50] with default parameters were used for alignment: for the four rRNA genes (nucleotide), the Q-INS-i model, considering secondary RNA structure, was utilized; for the 13 protein-coding genes, in amino acid format, the G-INS-I model was used. The 17 separate alignments were edited manually using Mesquite v3.1 [51]. Ambiguously aligned characters were removed from each alignment using Gblocks [52] with least stringent parameters.

The single-gene alignments were concatenated using the catfasta2phyml perl script (https://github.com/nylander/catfasta2phyml) into three separate datasets: (i) rRNA operon (two rRNA genes; 18 s and 28 s), (ii) mitogenome (two rRNA genes; rrnS and rrnL and 13 protein coding genes), and finally (iii) rRNA+mitogenome (four rRNA genes and 13 protein coding genes). Each dataset was inferred with and without the outgroup. The alignments (both masked and unmasked) have been made freely available through the first author’s ResearchGate page (https://www.researchgate.net/home) and through Dryad (10.5061/dryad.7pvmcvdpn).

### Phylogenetic reconstruction

Maximum likelihood (ML) phylogenetic analyses were carried out for each single gene using the “AUTO” parameter in RAxML v8.0.26 [53] to establish the evolutionary model with the best fit. The general time reversible (GTR + G) is the preferred model for the four rRNA genes (18 s, 28 s, rrnS and rrnL), and MtZoa+G for all but one of the 13 protein coding genes, with MtArt+G being preferred for *atp8*. The concatenated datasets, divided into gene partitions (above), each with its own separate gamma distribution were analysed using RAxML; The topology with the highest likelihood score of 100 heuristic searches was chosen. Bootstrap values were calculated from 500 pseudo-replicates. Bayesian inference (BI) was performed using a modified version of MrBayes v3.2 [54] incorporating the MtArt and MtZoa evolutionary models (https://github.com/astanabe/mrbayes5d). The datasets were executed, as before, with genes partitions under their separate gamma distribution. Two independent runs, each with three heated and one cold Markov Chain Monte Carlo (MCMC) chain, were initiated from a random starting tree. The MCMC chains were run for 20,000,000 generations with trees sampled every 1000th generation. The posterior probabilities and mean marginal likelihood values of the trees were calculated after the burn-in phase. The average standard deviation of split frequencies between the two runs were < 0.01, indicating the convergence of the MCMC chains.

Congruence between the topological signal of the bryozoan rRNA operon (Additional file 1: Figure S1) and mitogenome (Additional file 1: Figure S2) ingroup trees was tested, to support their concatenation, using *I*_*cong*_ index [55].

As we have samples that belong to the same morphological species, as well as those that are nominally different but closely related, we were interested in exploring criteria for genetic similarity as used in other taxa [56, 57] to objectively delimit morphogenera and -species in our dataset. Hence, we ran Cd-hit v4.6.4 [58], a clustering software that groups samples according to sequence identity, with thresholds of 0.99, 0.98, 0.97, 0.95 and 0.90 on the DNA barcoding regions 18 s V4 [59], 28 s D1-D2 [60], and *cox1* [56]. Due to its higher interspecies sequence variability, *cox1* was additionally tested with identity thresholds set to 0.80.

## Supplementary information

**Additional file 1: **Supporting Figures for Orr et al. Genome skimming the bryozoan tree: lessons from a widespread family, Adeonidae. **Figure S1.** The phylogeny of adeonids based on two nuclear rRNA genes. Maximum likelihood topology of 35 adeonid ingroup taxa with 5478 nucleotide characters inferred using RAxML. The numbers on the internal nodes are ML bootstrap values (RAxML). The scale bare represents substitutions per site. **Figure S2.** The phylogeny of adeonids based on 15 mitochondrial genes. Maximum likelihood topology of 35 adeonid ingroup taxa with 5053 nucleotide and amino acid characters inferred using RAxML. The numbers on the internal nodes are ML bootstrap values (RAxML). The scale bare represents substitutions per site. **Figure S3.** The phylogeny of adeonids based on 17 genes. Maximum likelihood topology of 35 adeonid ingroup taxa and eight outgroup taxa with 10,259 nucleotide and amino acid characters inferred using RAxML. The numbers on the internal nodes are ML bootstrap values (RAxML). Note that data from the outgroup taxa are from NCBI (see Additional file 2: **Table S1**). The scale bare represents substitutions per site. **Figure S4.** Adeonid sequence identity for three barcode regions: The topology is identical to that depicted in Fig. 1. The figure shows the inferred adeonid ingroup phylogeny with clustering at varying levels of sequence identity using Cd-hit. 18 s V4 region (99, 98 and 97% cluster identity) in blue, 28 s D1-D2 regions (99, 97 and 90% cluster identity) in yellow, and *cox1* in (99 and 83% cluster identity) green. The coloured bars represent the taxa that clustered together at the varying identities, with dots indicating taxa that remained as solitary clusters. The scale bare represents substitutions per site.

**Additional file 2.** Expanded overview of metadata associated with the samples.

**Additional file 3.** Scanning Electron Micrographs (SEMs) of dried samples.

## Data Availability

All sequences produced in this study have been deposited in the NCBI database under the accessions MK894342-MK894410 and MN131144-MN131187. In addition, the alignments used for phylogenetic inference have been made freely available through the authors ResearchGate pages (https://www.researchgate.net/home) and Dryad (10.5061/dryad.7pvmcvdpn).

## Abbreviations

atp6: Mitochondrially Encoded ATP Synthase Membrane Subunit 6
atp8: Mitochondrially Encoded ATP Synthase Membrane Subunit 8
BS: Bootstrap
cob: cytochrome b
cox1: Cytochrome c oxidase I
cox2: Cytochrome c oxidase 2
cox3: Cytochrome c oxidase 3
DNA: Deoxyribonucleic acid
LSU (28 s): large subunit
MCMC: Markov Chain Monte Carlo
nad1: NADH dehydrogenase subunit 1
nad2: NADH dehydrogenase subunit 2
nad3: NADH dehydrogenase subunit 3
nad4: NADH dehydrogenase subunit 4
nad4l: NADH dehydrogenase subunit 4 L
nad5: NADH dehydrogenase subunit 5
nad6: NADH dehydrogenase subunit 6
NCBI: The National Center for Biotechnology Information
PCR: Polymerase chain reaction
PE: Paired end
PP: Posterior probability
RAxML: Randomized Axelerated Maximum Likelihood
rRNA: Ribosomal ribonucleic acid
rrnL: mitochondrial large ribosomal subunit
rrns: mitochondrial small ribosomal subunit
s.l.: sensu lato
s.s.: sensu stricto
SEM: Scanning electron microscopy
SSU (18 s): Small subunit
