# Supplementary material for: A genome-skimmed phylogeny of a widespread bryozoan family, Adeonidae
Source: BMC Evol Biol. 2019 Dec 27;19:235. doi: 10.1186/s12862-019-1563-4 (PMC6935126; doi:10.1186/s12862-019-1563-4)
Supplement: Supplementary file 1 — Additional file 1: Supporting Figures for Orr et al. Genome skimming the bryozoan tree: lessons from a widespread family, Adeonidae. Figure S1. The phylogeny of adeonids based on two nuclear rRNA genes. Maximum likelihood topology of 35 adeonid ingroup taxa with 5478 nucleotide characters inferred using RAxML. The numbers on the internal nodes are ML bootstrap values (RAxML). The scale bare represents substitutions per site. Figure S2. The phylogeny of adeonids based on 15 mitochondrial genes. Maximum likelihood topology of 35 adeonid ingroup taxa with 5053 nucleotide and amino acid characters inferred using RAxML. The numbers on the internal nodes are ML bootstrap values (RAxML). The scale bare represents substitutions per site. Figure S3. The phylogeny of adeonids based on 17 genes. Maximum likelihood topology of 35 adeonid ingroup taxa and eight outgroup taxa with 10,259 nucleotide and amino acid characters inferred using RAxML. The numbers on the internal nodes are ML bootstrap values (RAxML). Note that data from the outgroup taxa are from NCBI (see Additional file 2: Table S1). The scale bare represents substitutions per site. Figure S4. Adeonid sequence identity for three barcode regions: The topology is identical to that depicted in Fig. 1. The figure shows the inferred adeonid ingroup phylogeny with clustering at varying levels of sequence identity using Cd-hit. 18 s V4 region (99, 98 and 97% cluster identity) in blue, 28 s D1-D2 regions (99, 97 and 90% cluster identity) in yellow, and cox1 in (99 and 83% cluster identity) green. The coloured bars represent the taxa that clustered together at the varying identities, with dots indicating taxa that remained as solitary clusters. The scale bare represents substitutions per site. [file 12862_2019_1563_MOESM1_ESM.docx]

**Supporting Figures for Orr et al. Genome skimming the bryozoan tree: lessons from a widespread family, Adeonidae**

**Figure S1. The phylogeny of adeonids based on two nuclear rRNA genes.** Maximum likelihood topology of 35 adeonid ingroup taxa with 5478 nucleotide characters inferred using RAxML. The numbers on the internal nodes are ML bootstrap values (RAxML). The scale bare represents substitutions per site.

**Figure S2. The phylogeny of adeonids based on 15 mitochondrial genes.** Maximum likelihood topology of 35 adeonid ingroup taxa with 5053 nucleotide and amino acid characters inferred using RAxML. The numbers on the internal nodes are ML bootstrap values (RAxML). The scale bare represents substitutions per site.

**Figure S3. The phylogeny of adeonids based on 17 genes.** Maximum likelihood topology of 35 adeonid ingroup taxa and eight outgroup taxa with 10259 nucleotide and amino acid characters inferred using RAxML. The numbers on the internal nodes are ML bootstrap values (RAxML). Note that data from the outgroup taxa are from NCBI (see Table S1). The scale bare represents substitutions per site.

**Figure S4. Adeonid sequence identity for three barcode regions:** The topology and is identical to that depicted in Fig. 1. The figure shows the inferred adeonid ingroup phylogeny with clustering at varying levels of sequence identity using Cd-hit. 18s V4 region (99, 98 and 97% cluster identity) in blue, 28s D1-D2 regions (99, 97 and 90% cluster identity) in yellow, and *cox1* in (99 and 83% cluster identity) green. The coloured bars represent the taxa that clustered together at the varying identities, with dots indicating taxa that remained as solitary clusters. The scale bare represents substitutions per site.
