# Supplementary material for: A genome-skimmed phylogeny of a widespread bryozoan family, Adeonidae
Source: BMC Evol Biol. 2019 Dec 27;19:235. doi: 10.1186/s12862-019-1563-4 (PMC6935126; doi:10.1186/s12862-019-1563-4)

BLEED 298

Adeona foliifera fascialis

NMV F 234 199
NHMO H 1411

Abrolhos,
West Australia
Australia

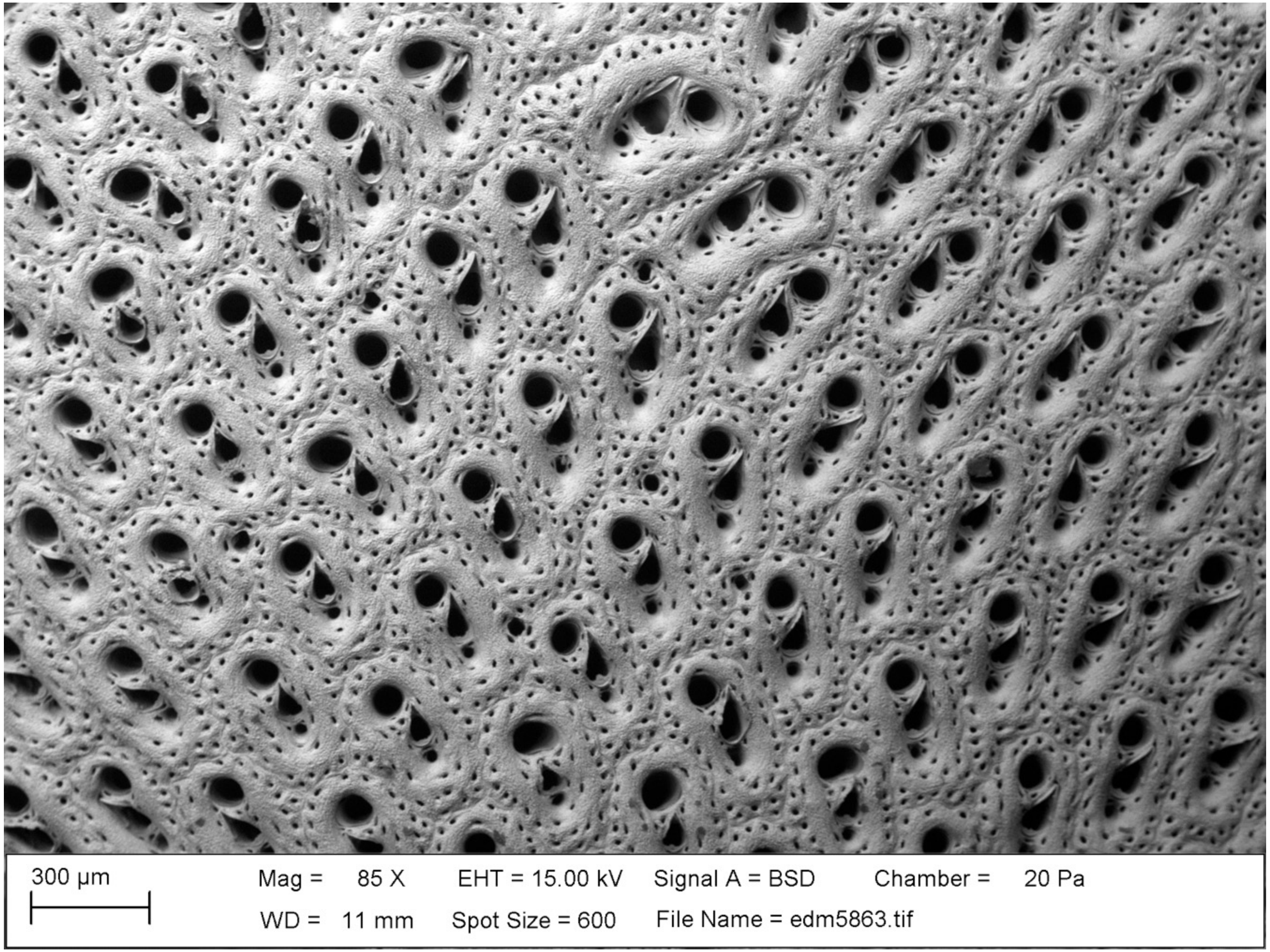

BLEED 297

Adeona foliifera fascialis

NMV F 234 198
NHMO H 1412

Off Carnarvon,
West Australia
Australia

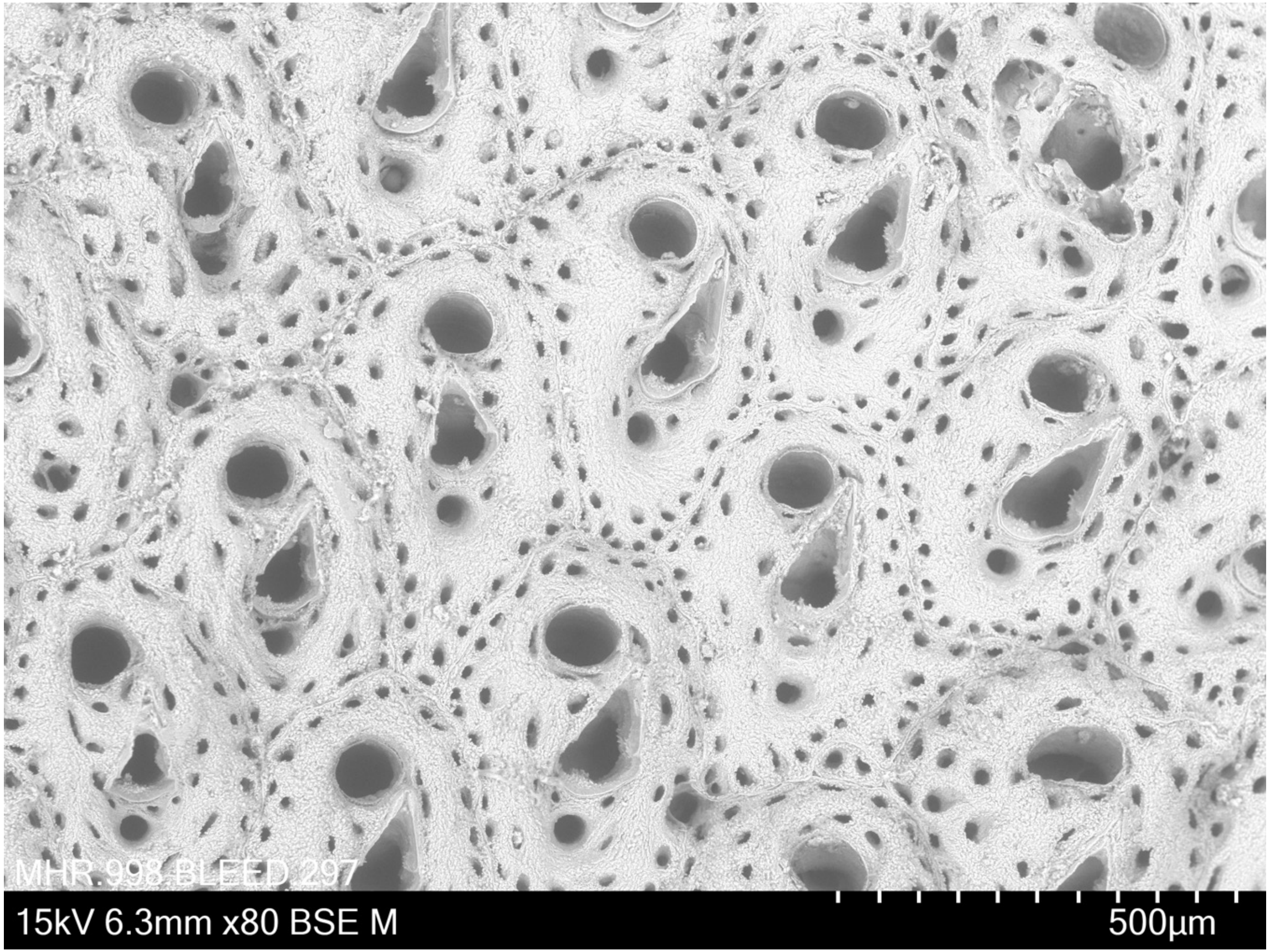

BLEED 746

Adeona sp. 1

NMV F 234 197
NHMO H 1414

off Bald Island
Australia

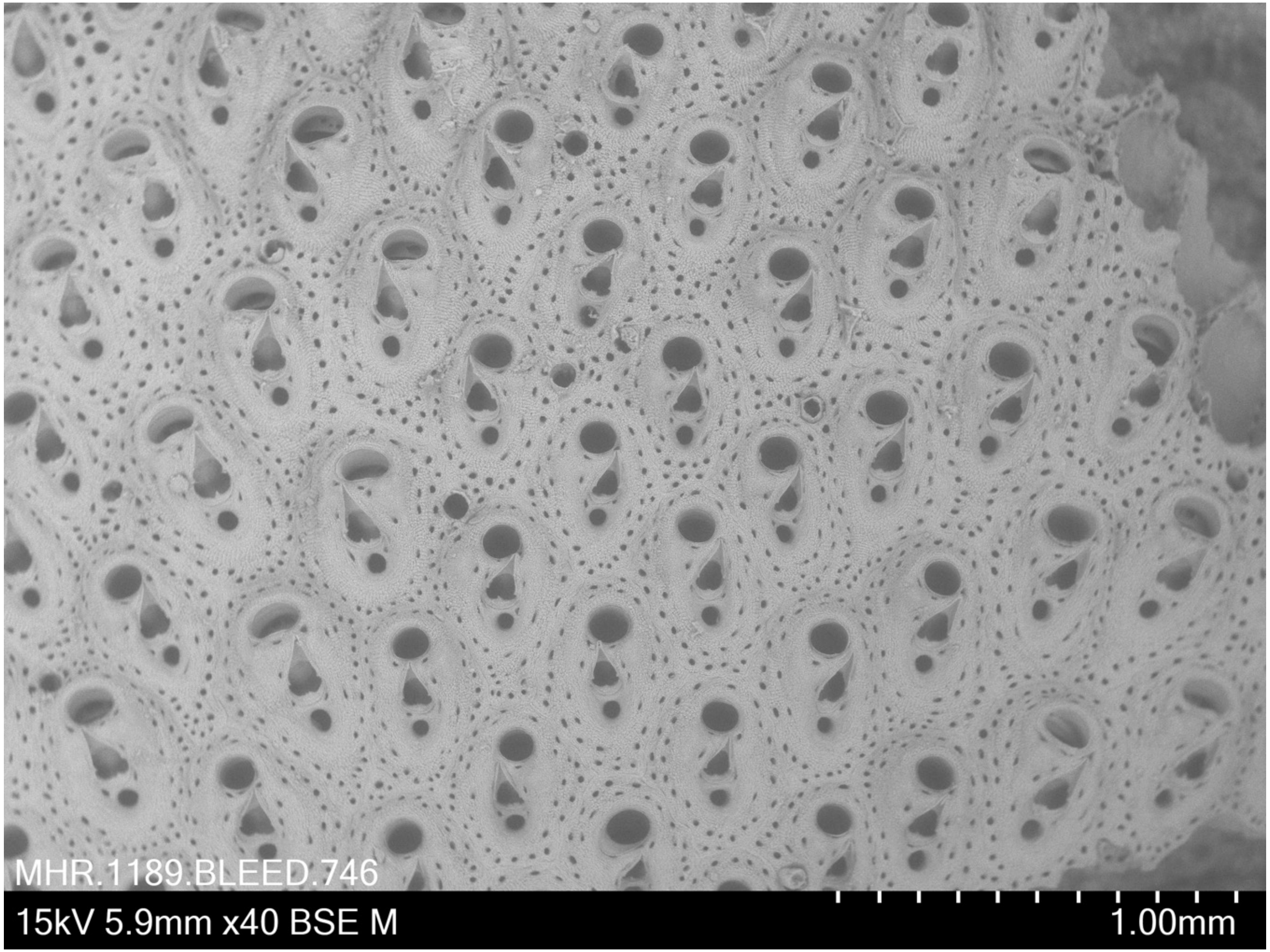

BLEED 749

Adeona sp. 1

NMV F 234 201
NHMO H 1413

off D'Entrecasteaux
Australia

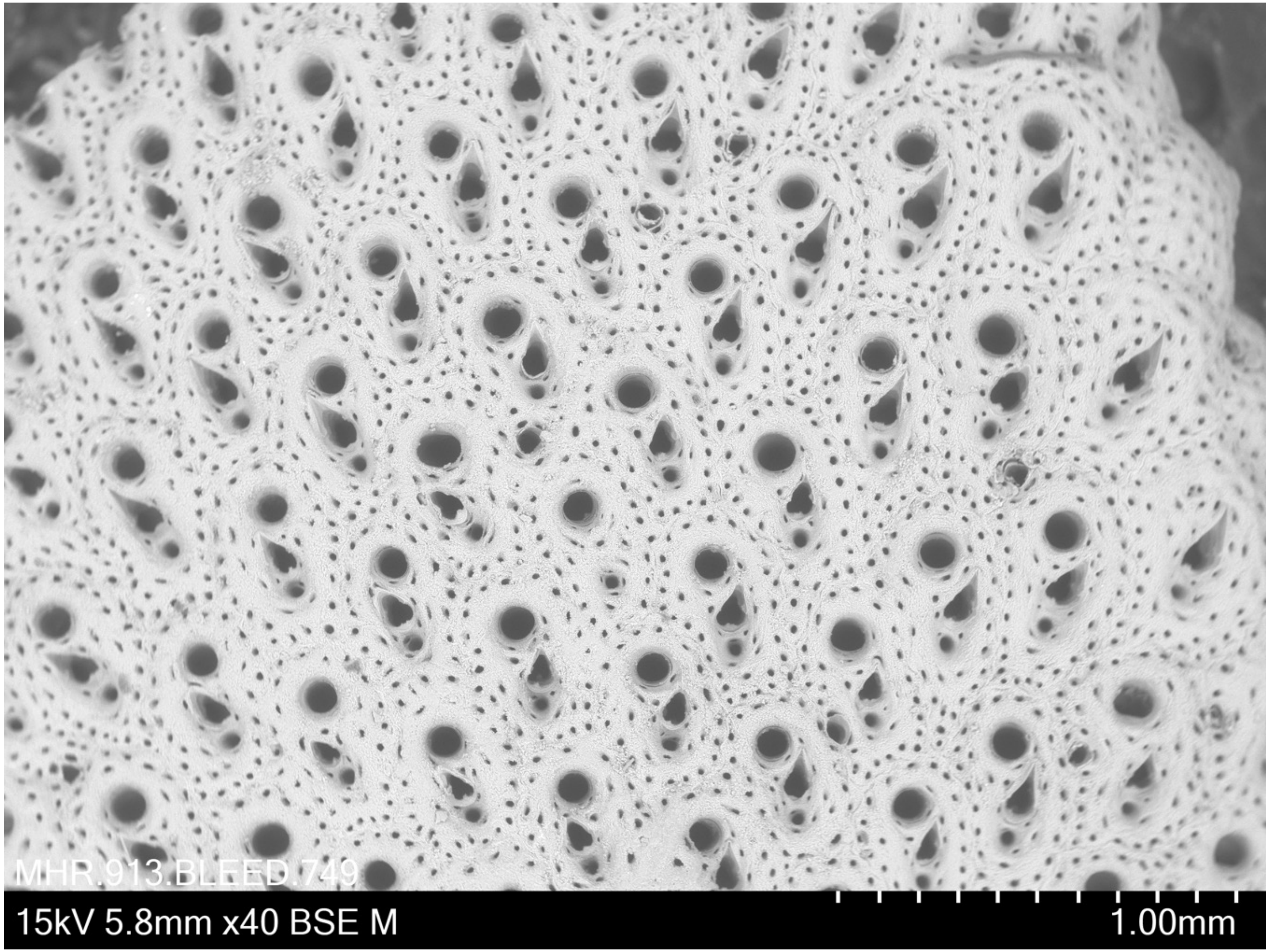

BLEED 293

Adeona sp. 2

NMV F 213 637
NHMO H 1415

off Albany
Australia

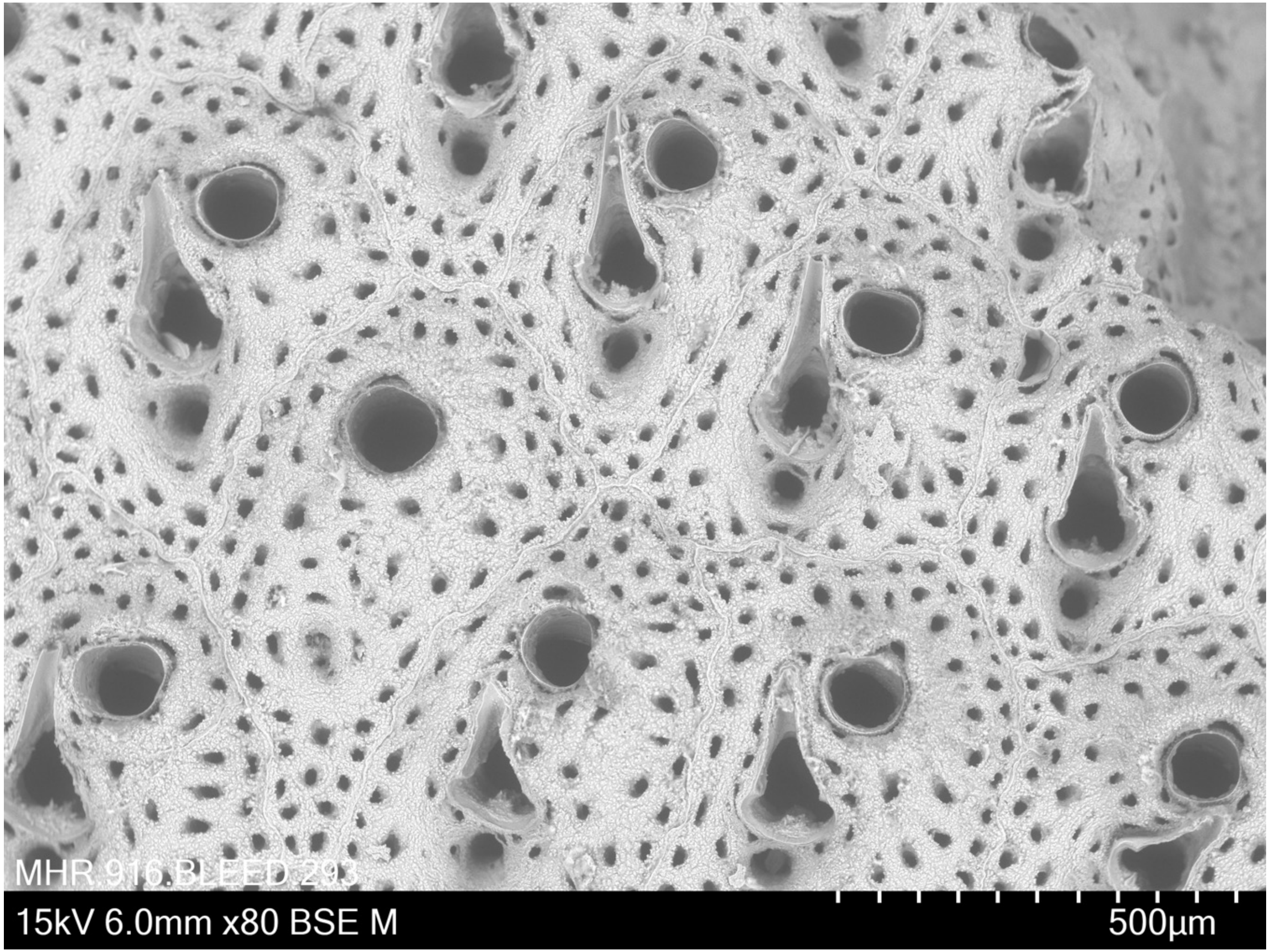

BLEED 295

Adeona sp. 2

NMV F 214 412
NHMO H 1416

off Kalbarri
Australia

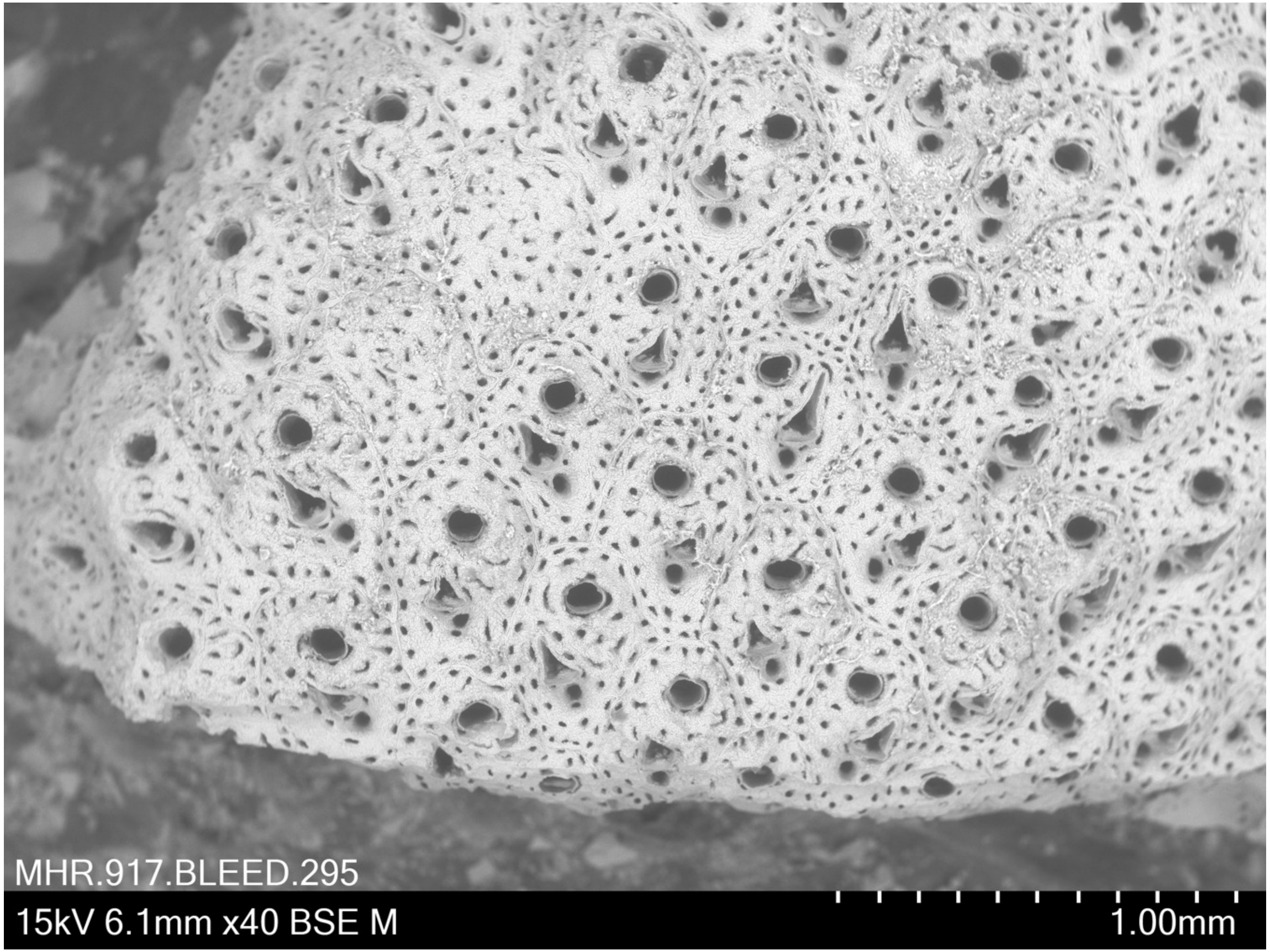

BLEED 292

Adeona sp. 3

NMV F 167487
NHMO H 1417

Northwestern
Australia

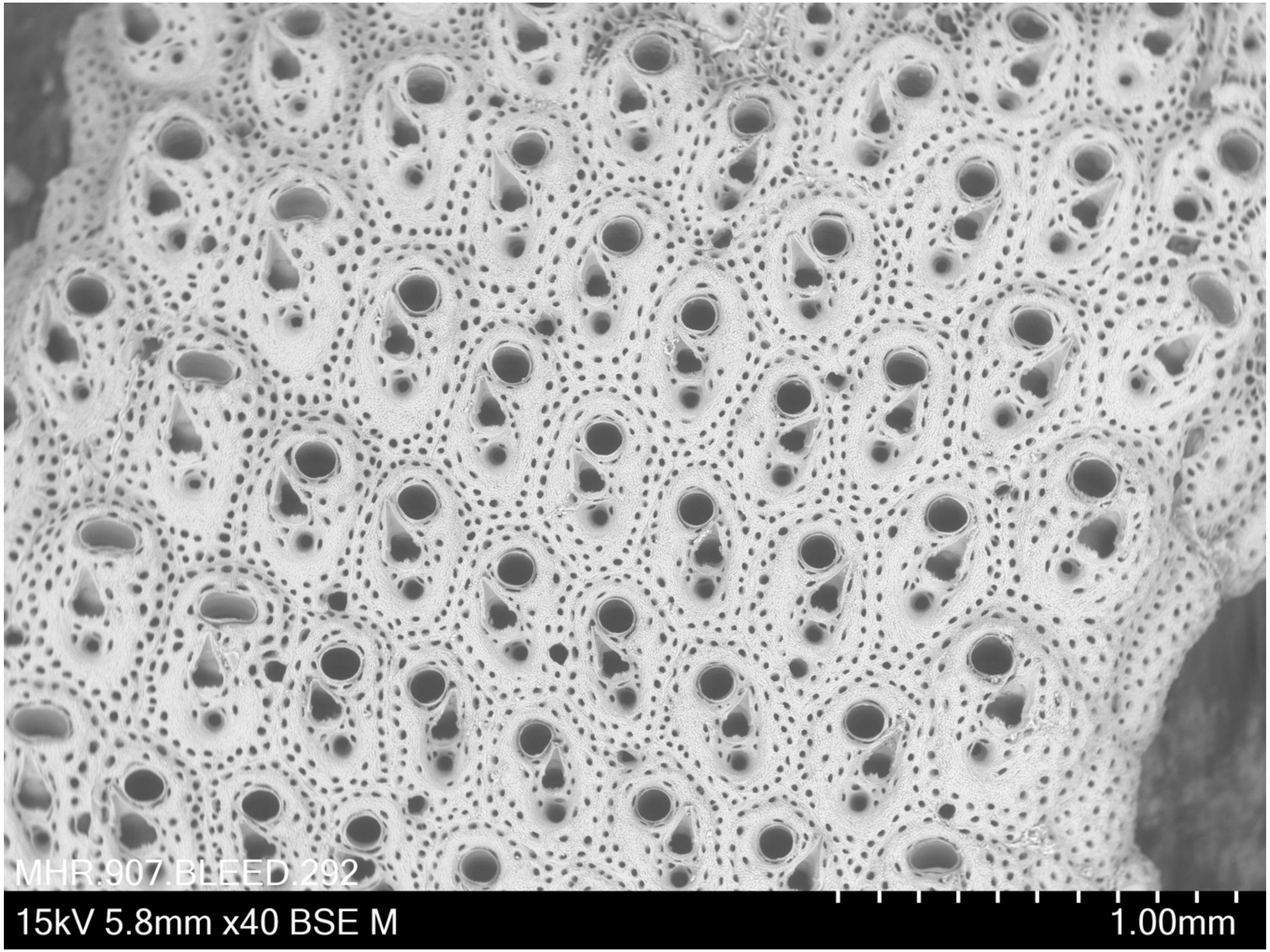

BLEED 294

Adeona sp. 4

NMV F 214 404
NHMO H 1418

Off Pt Hillier
Australia

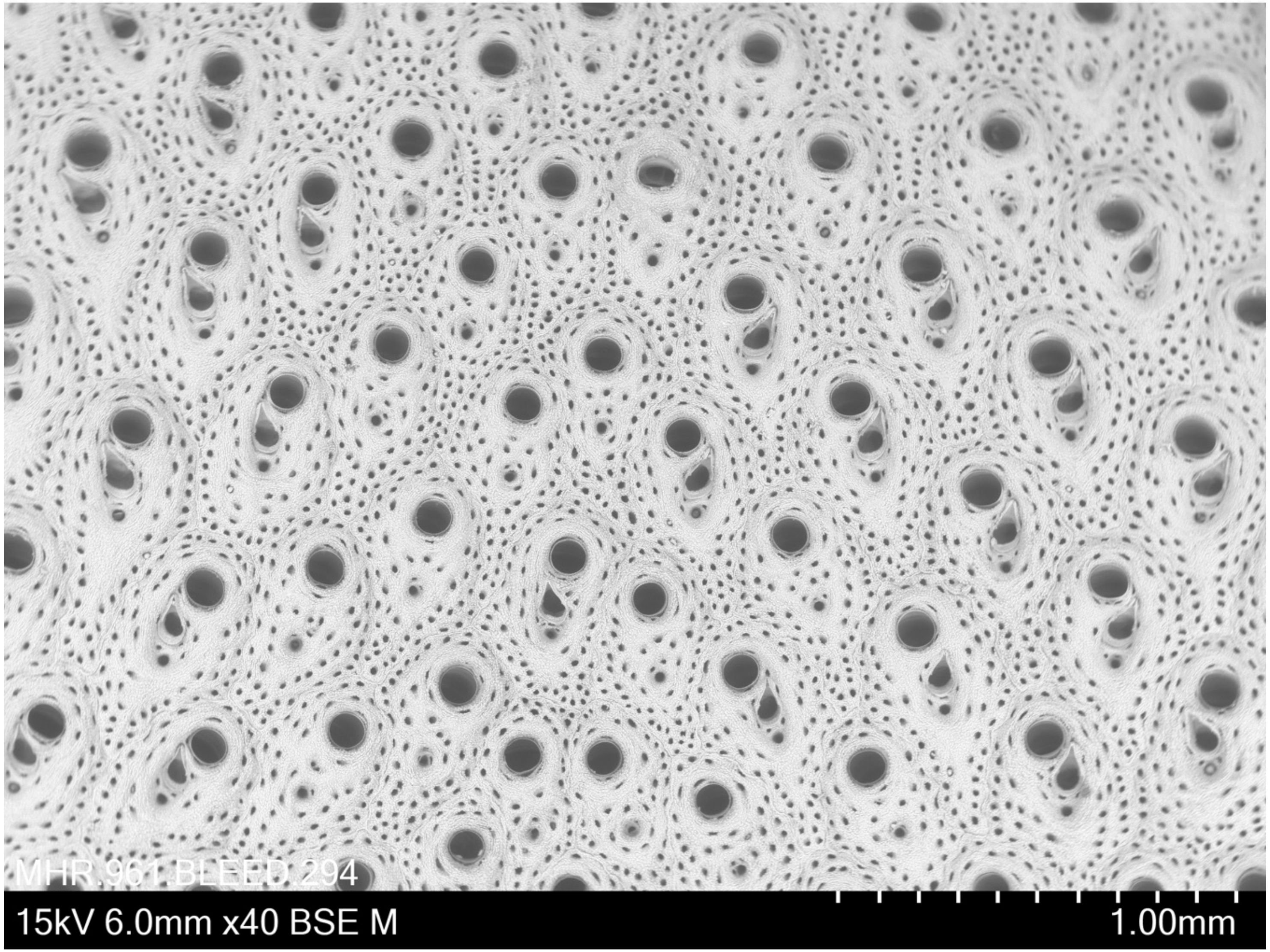

BLEED 438

Adeona sp. 5

WAM Z90464
NHMO H 1419

Camden Sound
Australia

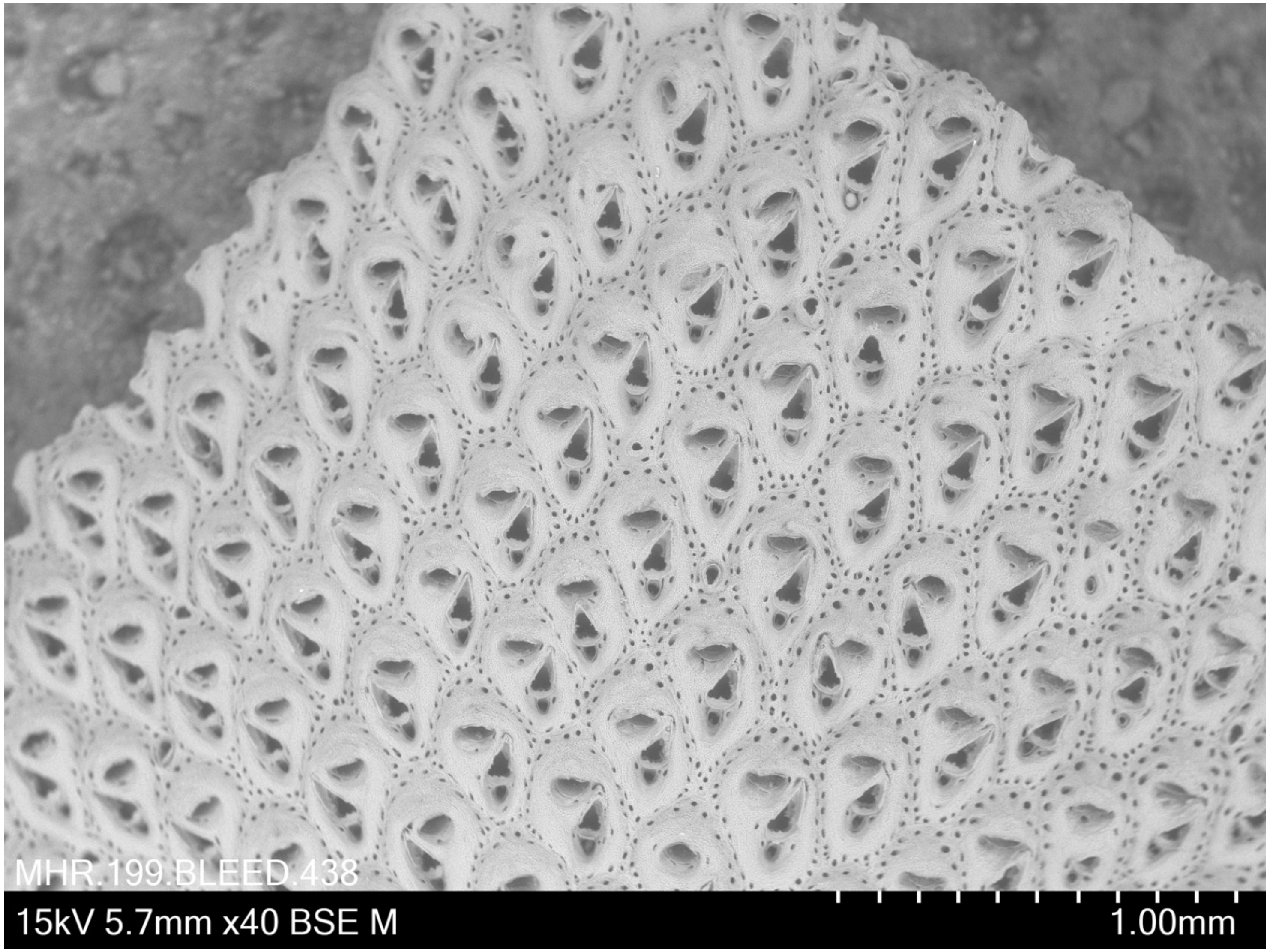

BLEED 444

Adeona sp. 5

WAM Z90466
NHMO H 1420

Camden Sound
Australia

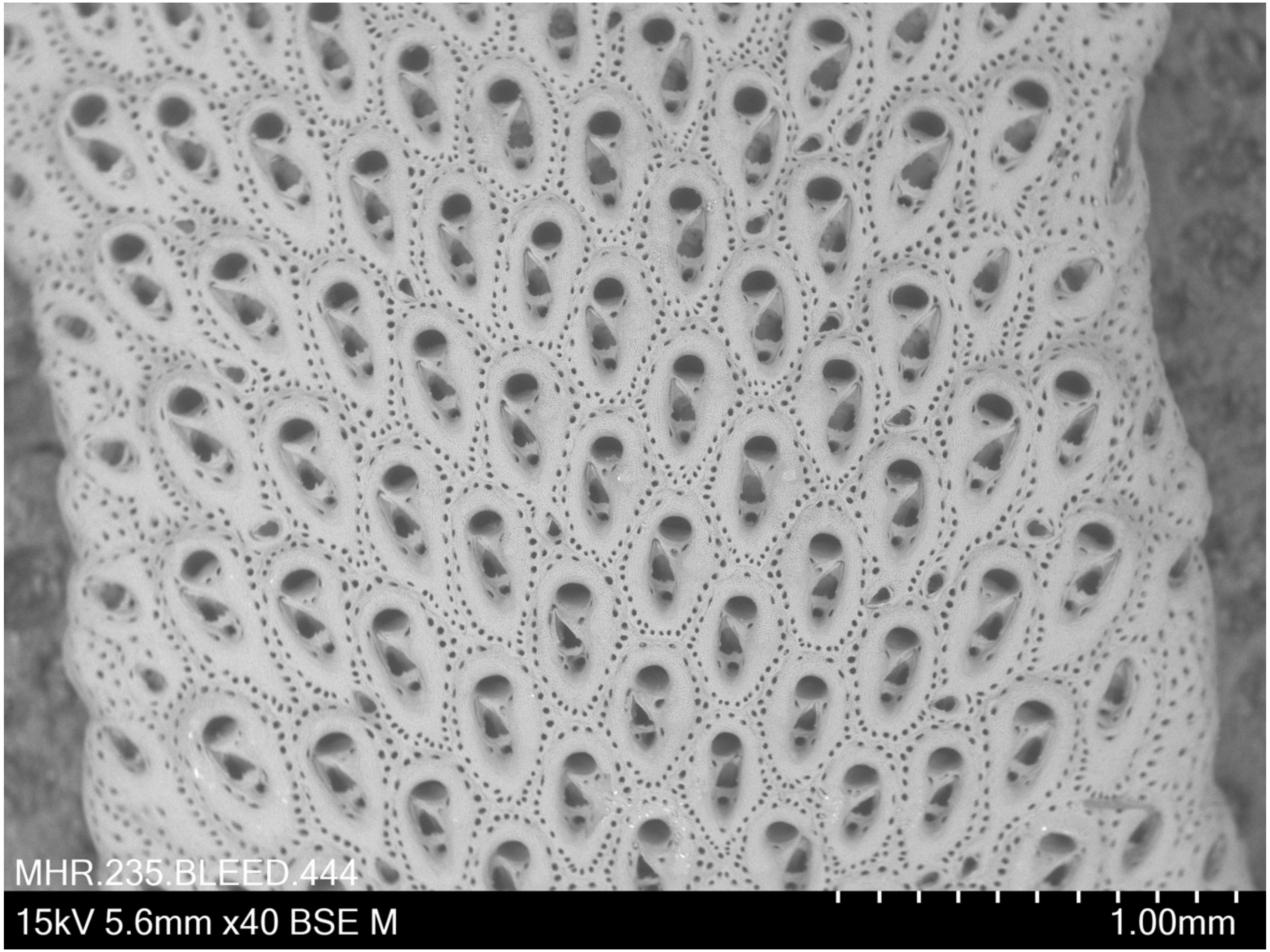

BLEED 49

Adeona japonica

NHMO H 1421

off Otsuchi Bay, Iwate Pref.
Japan

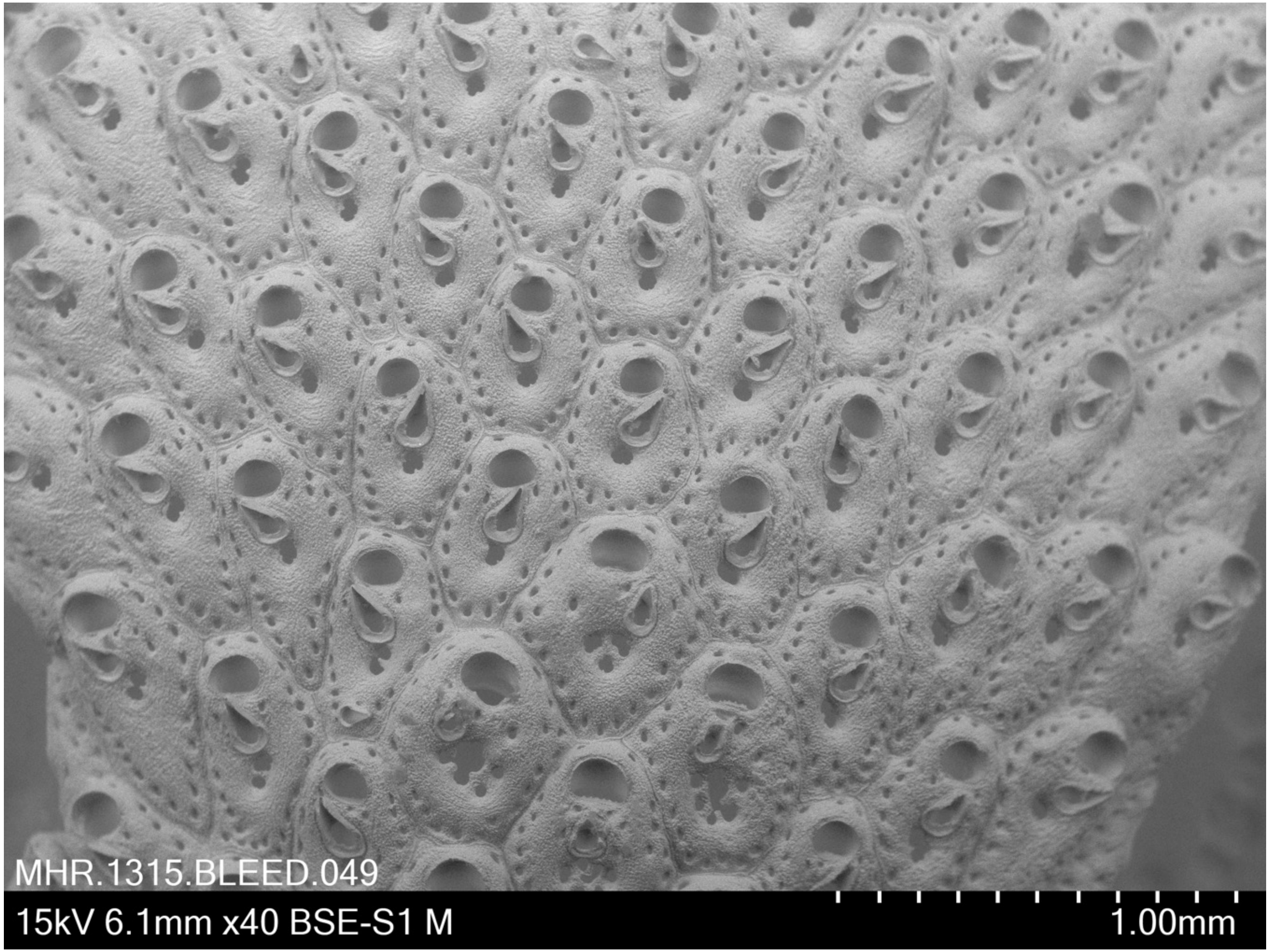

BLEED 303

Adeonellopsis sp. 1

NMV F 234 204
NHMO H 1422

off Bald Island
Australia

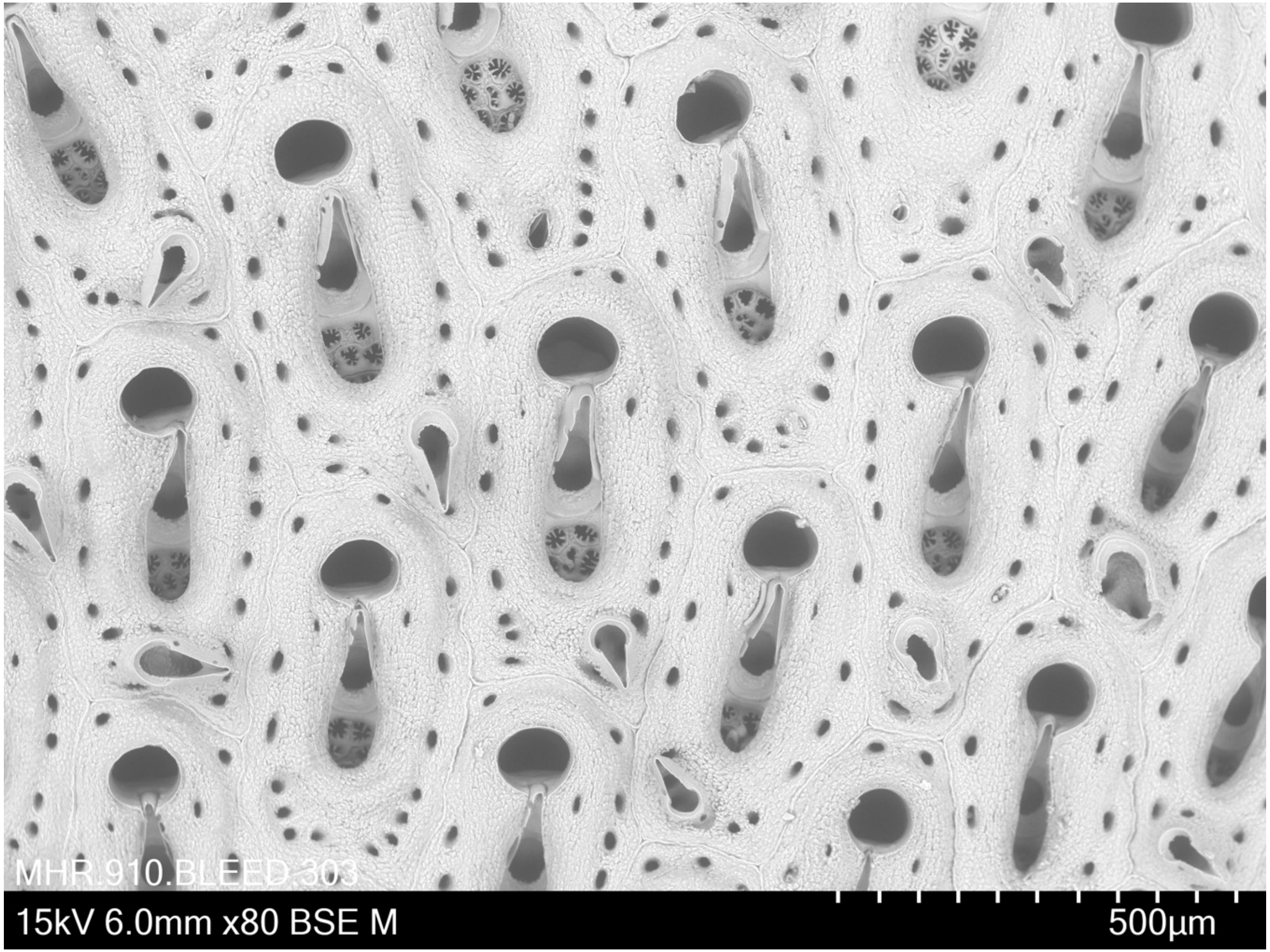

BLEED 301

Adeonellopsis sp.2

NMV F 214 422
NHMO H 1423

off Bald Island
Australia

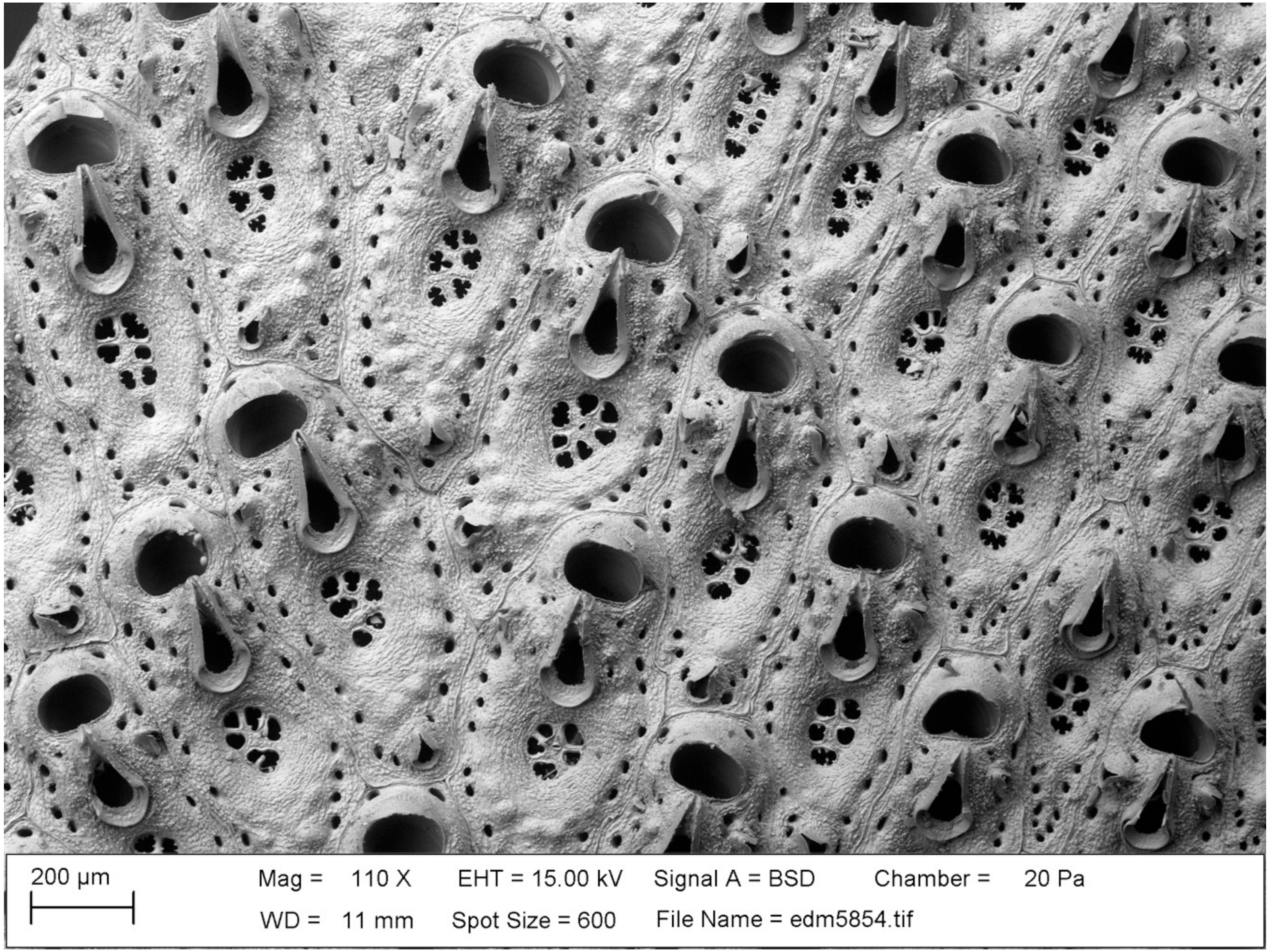

BLEED 300

Adeonellopsis cf. *australis*

NMV F 213 648
NHMO H 1425

off Albany
Australia

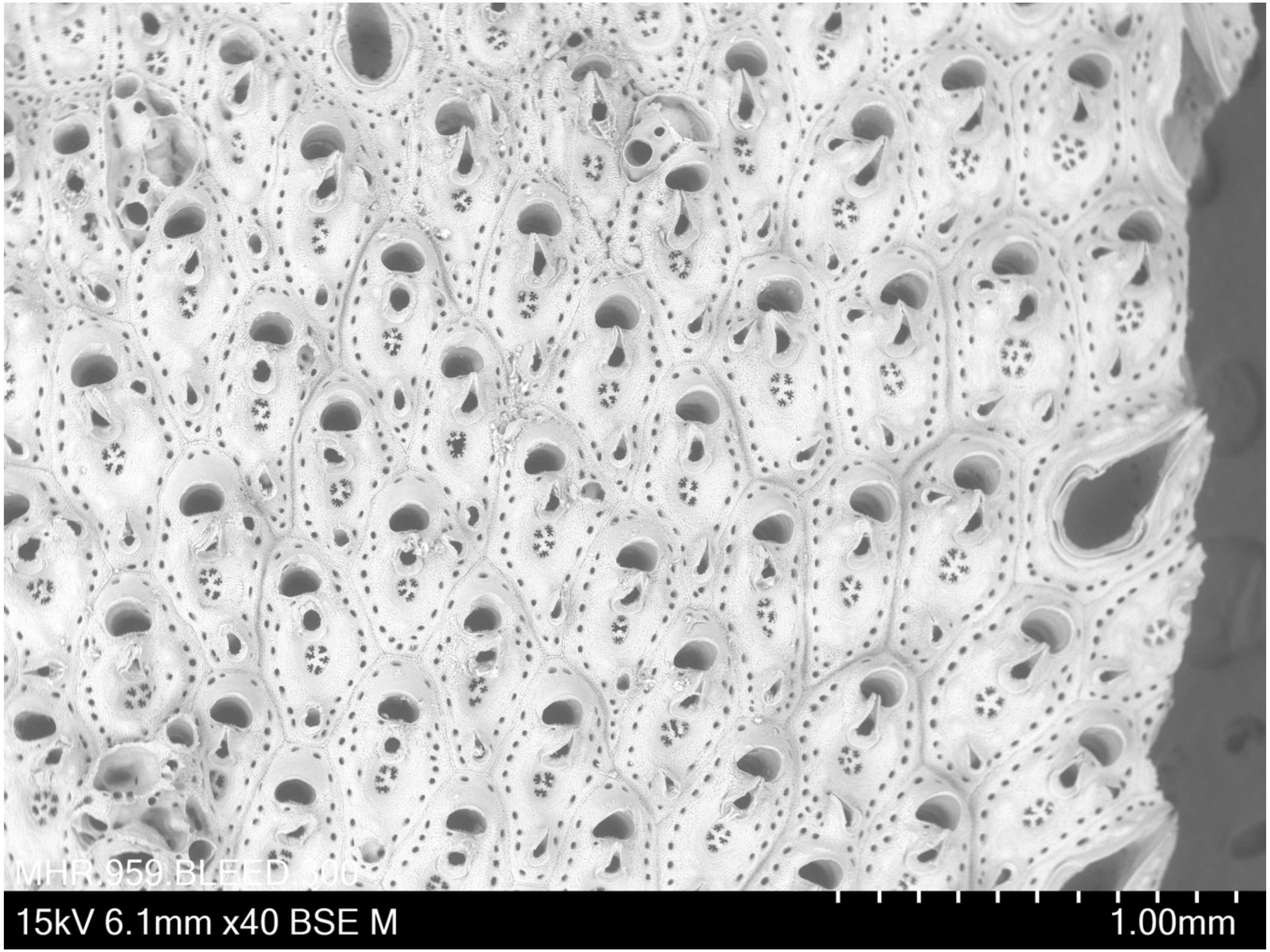

BLEED 306

Adeonellopsis sp.3

NMV F 234 206
NHMO H 1424

Shark Bay
Australia

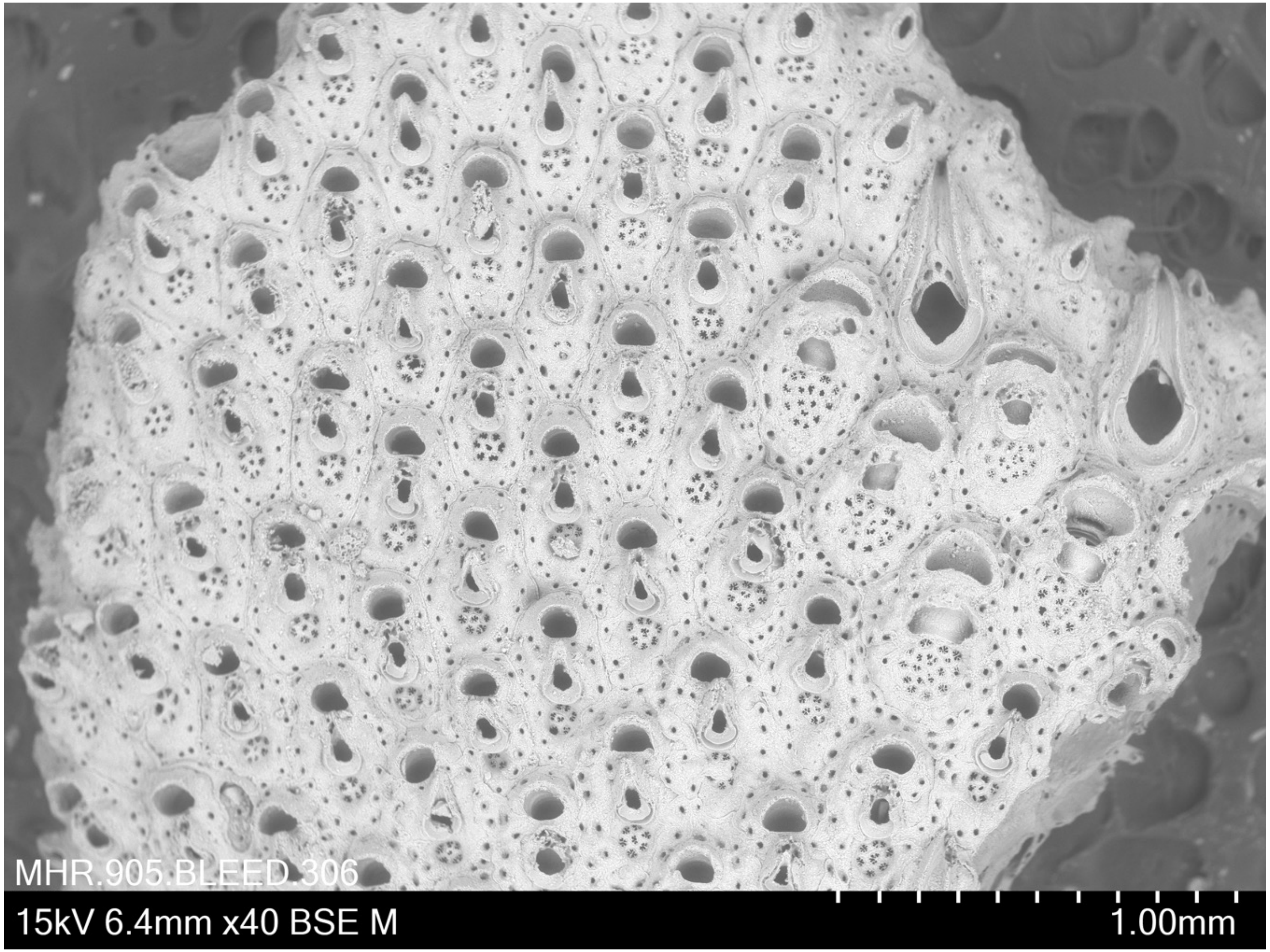

BLEED 344

Adeonellopsis sp. 4

NHMO H 1427

Snares
New Zealand

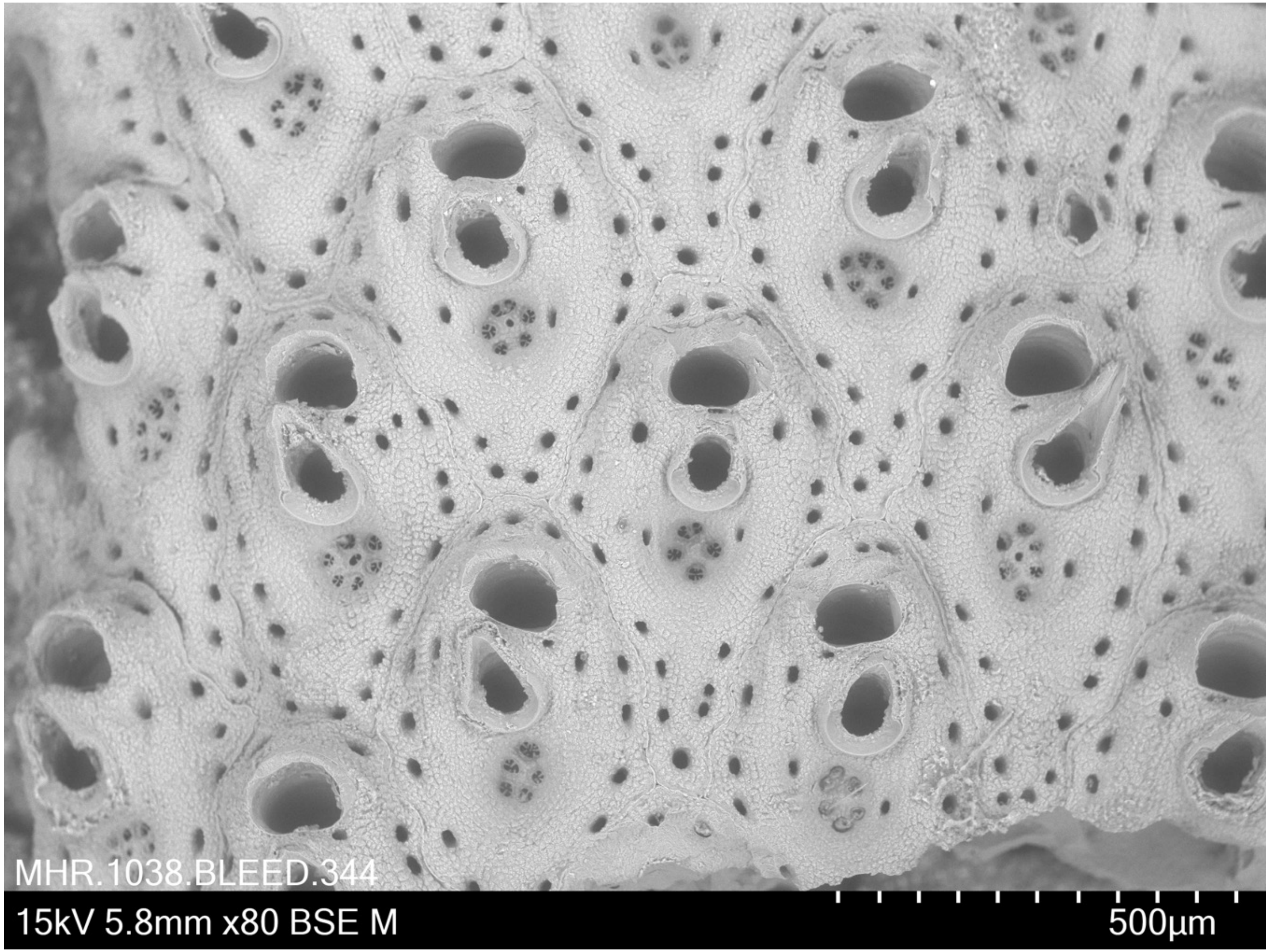

BLEED 48

Adeonellopsis sp. 4

NHMO H 1426

Snares
New Zealand

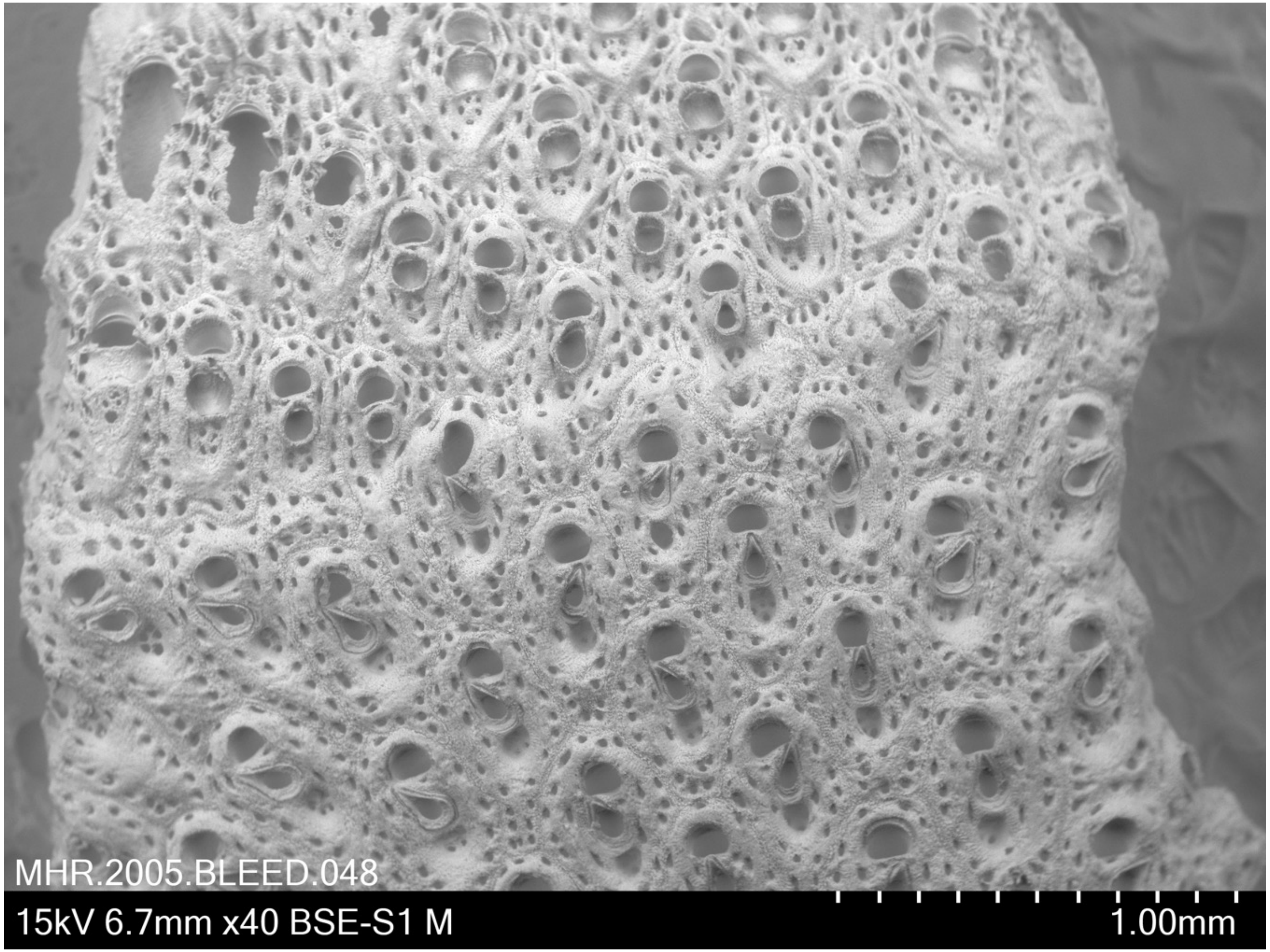

BLEED 750

Bracebridgia sp.

NMV F 234 203
NHMO H 1428

Off Albany
Australia

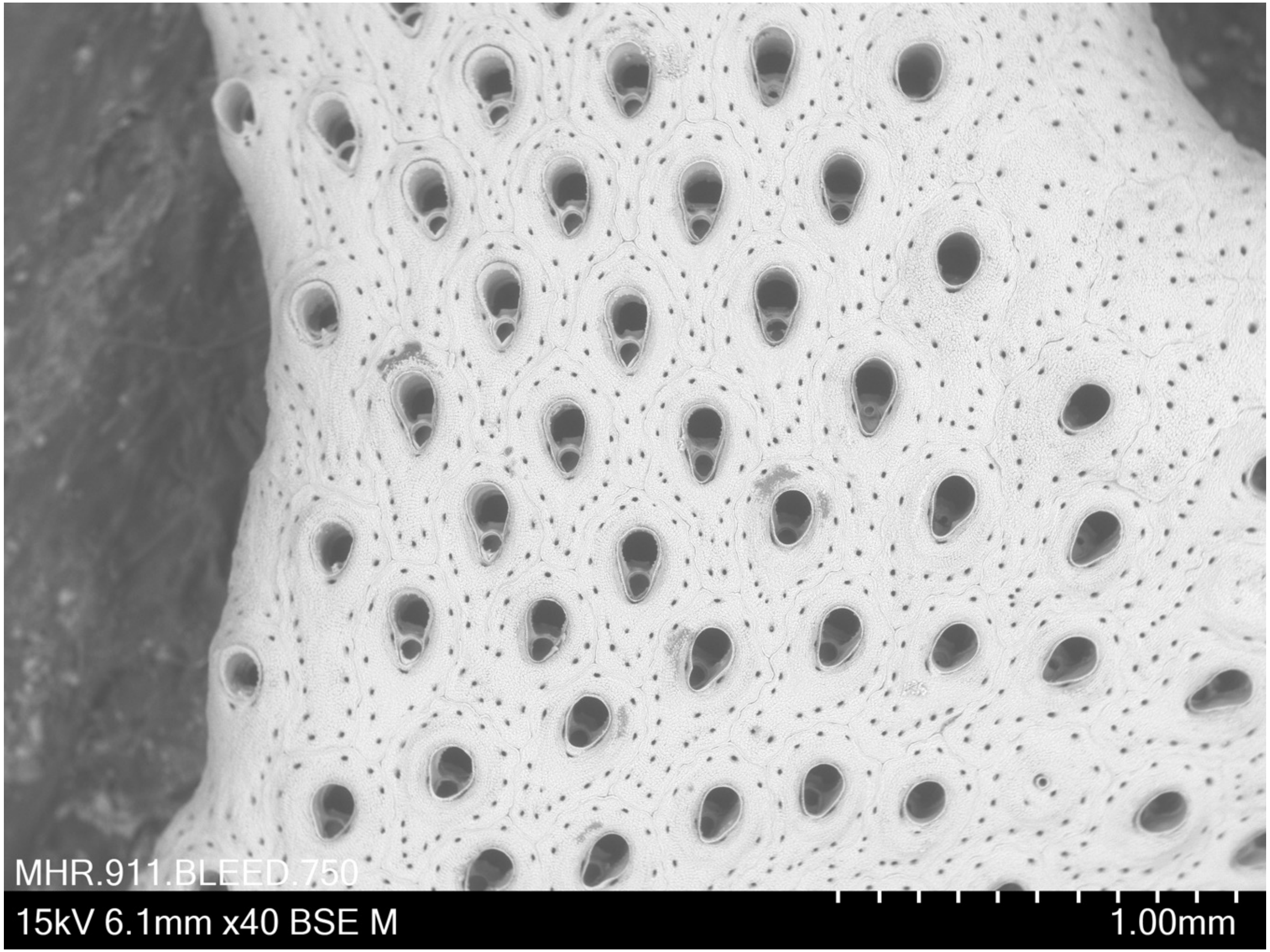

BLEED 305

Adeonella cf. *lichenoides*

NMV F 234 196
NHMO H 1434

Off Carnarvon
Australia

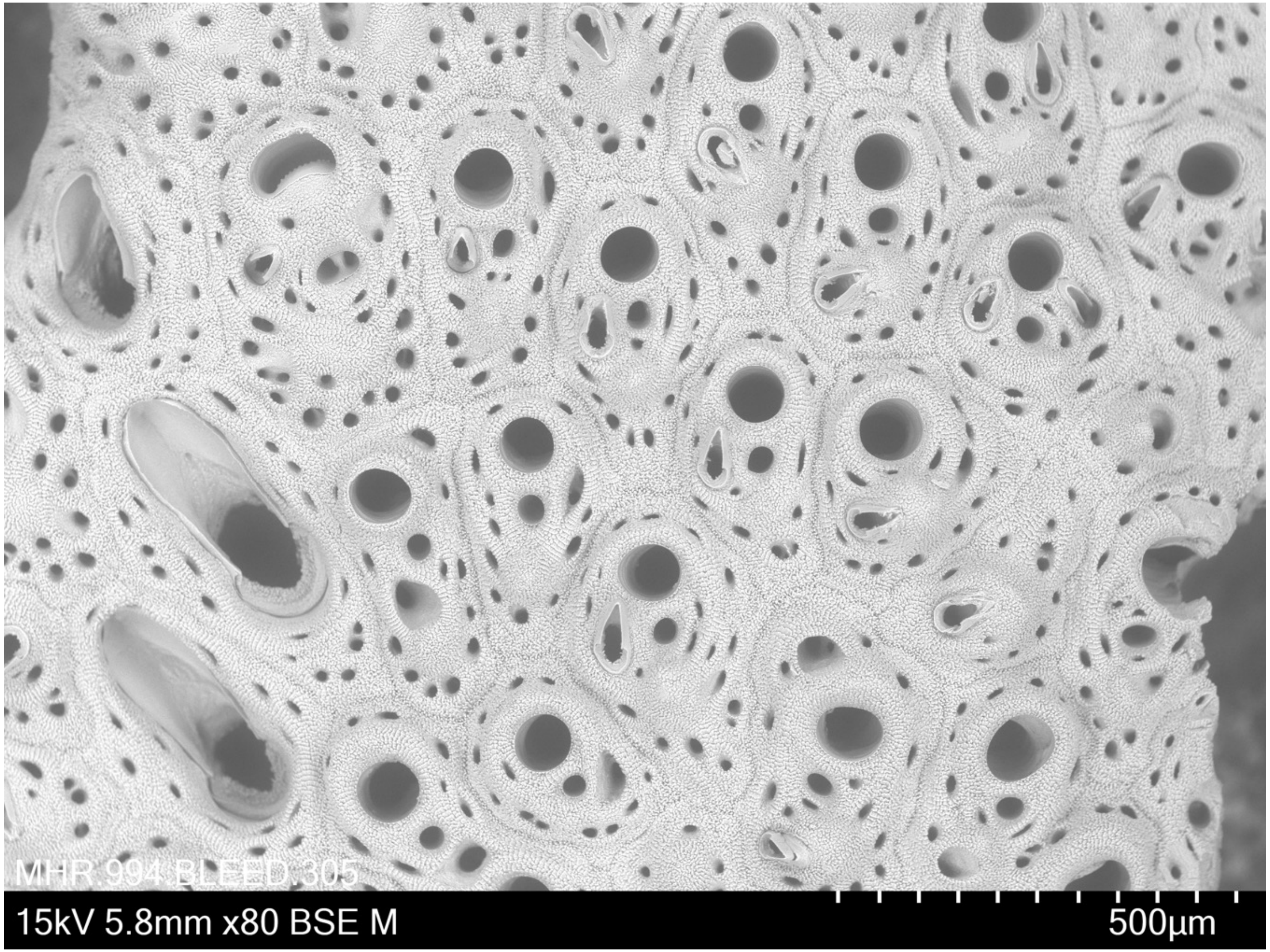

BLEED 439

Adeonella cf. *lichenoides*

WAM Z90866
NHMO H 1433

Eclipse Islands
Australia

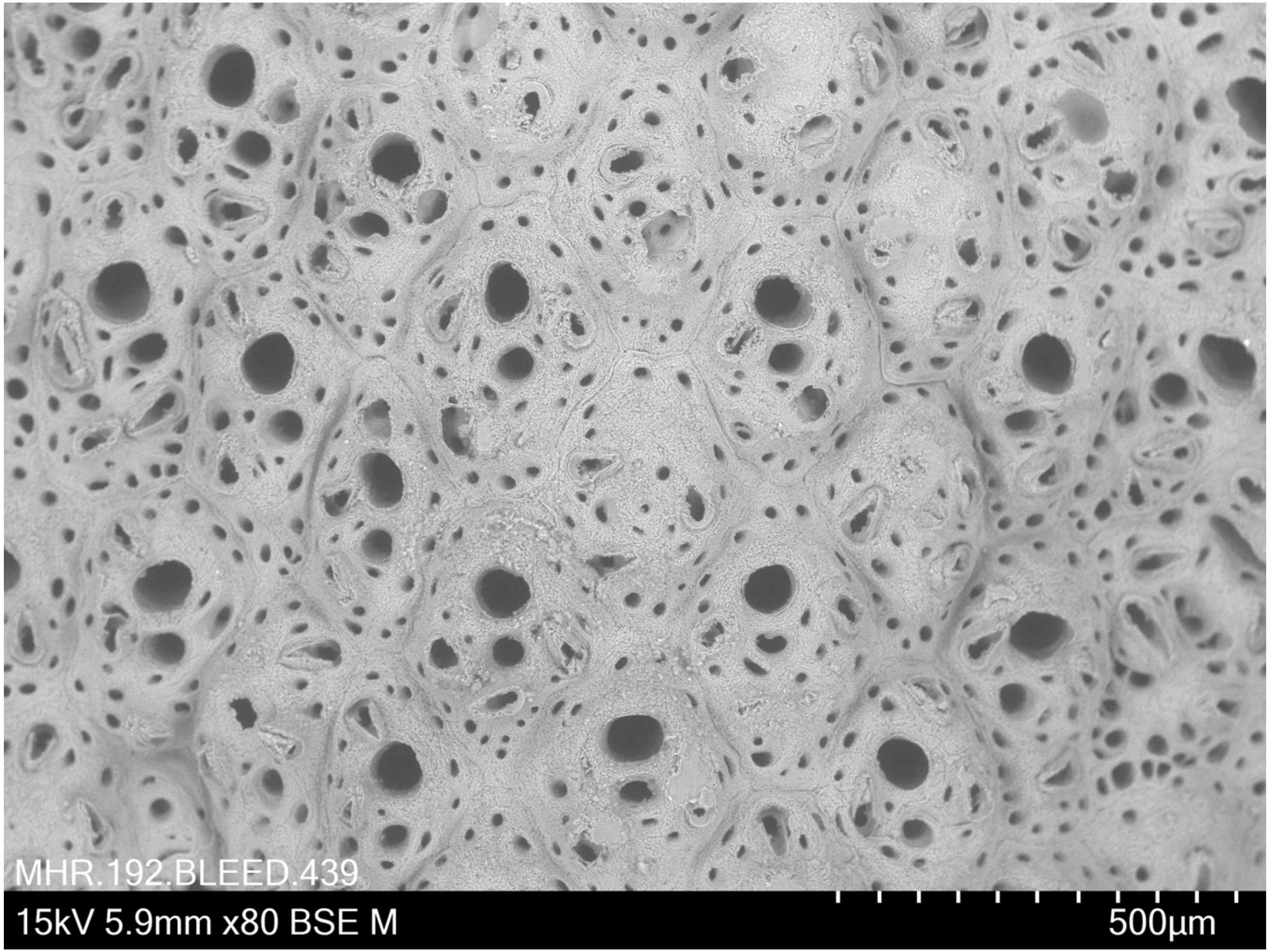

BLEED 429

Adeonella cf. *lichenoides*

WAM Z92433
NHMO H 1432

Maret Islands
Australia

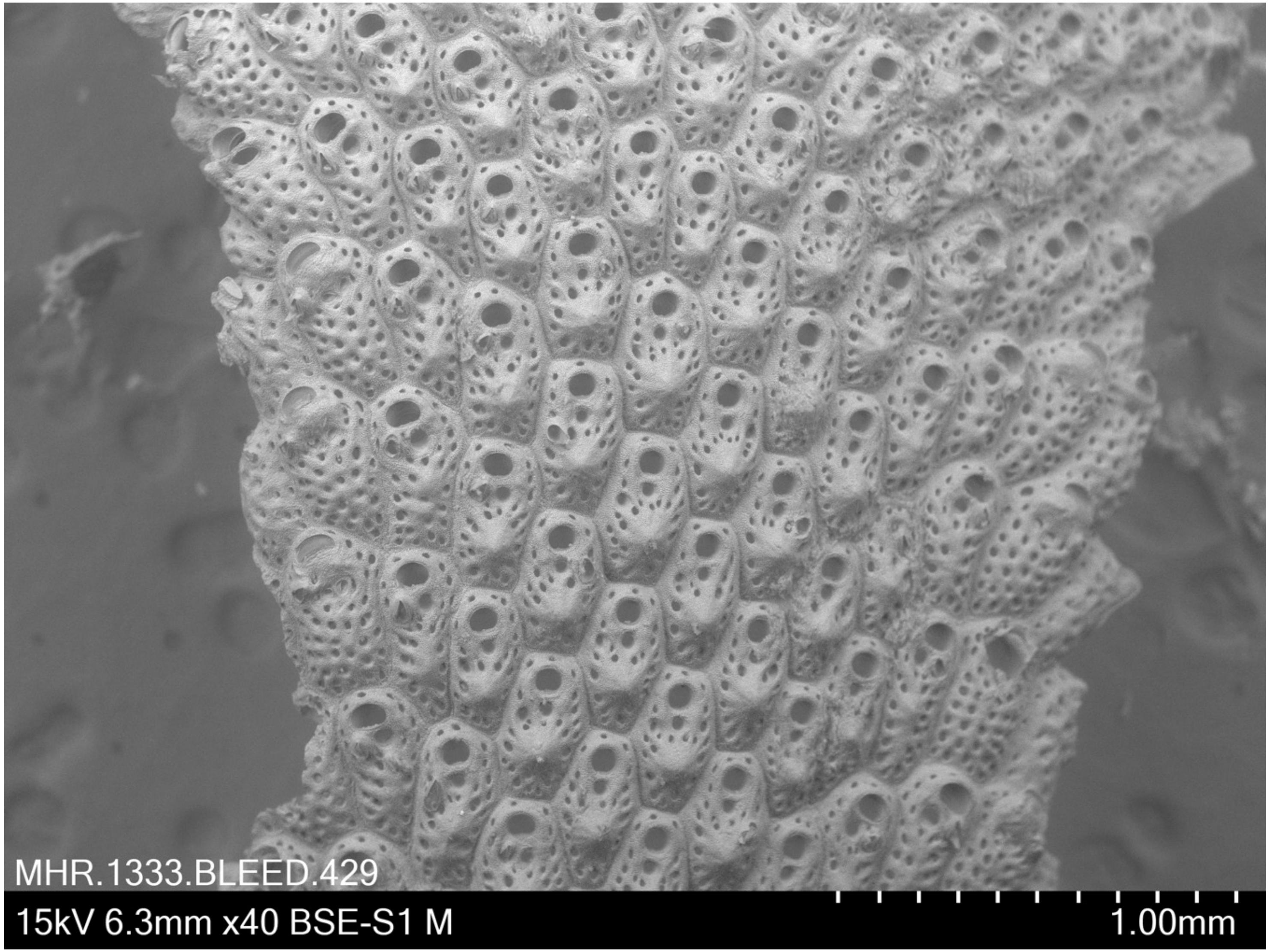

BLEED 416

Adeonella sp. 1

WAM Z90458
NHMO H 1435

Camden Sound
Australia

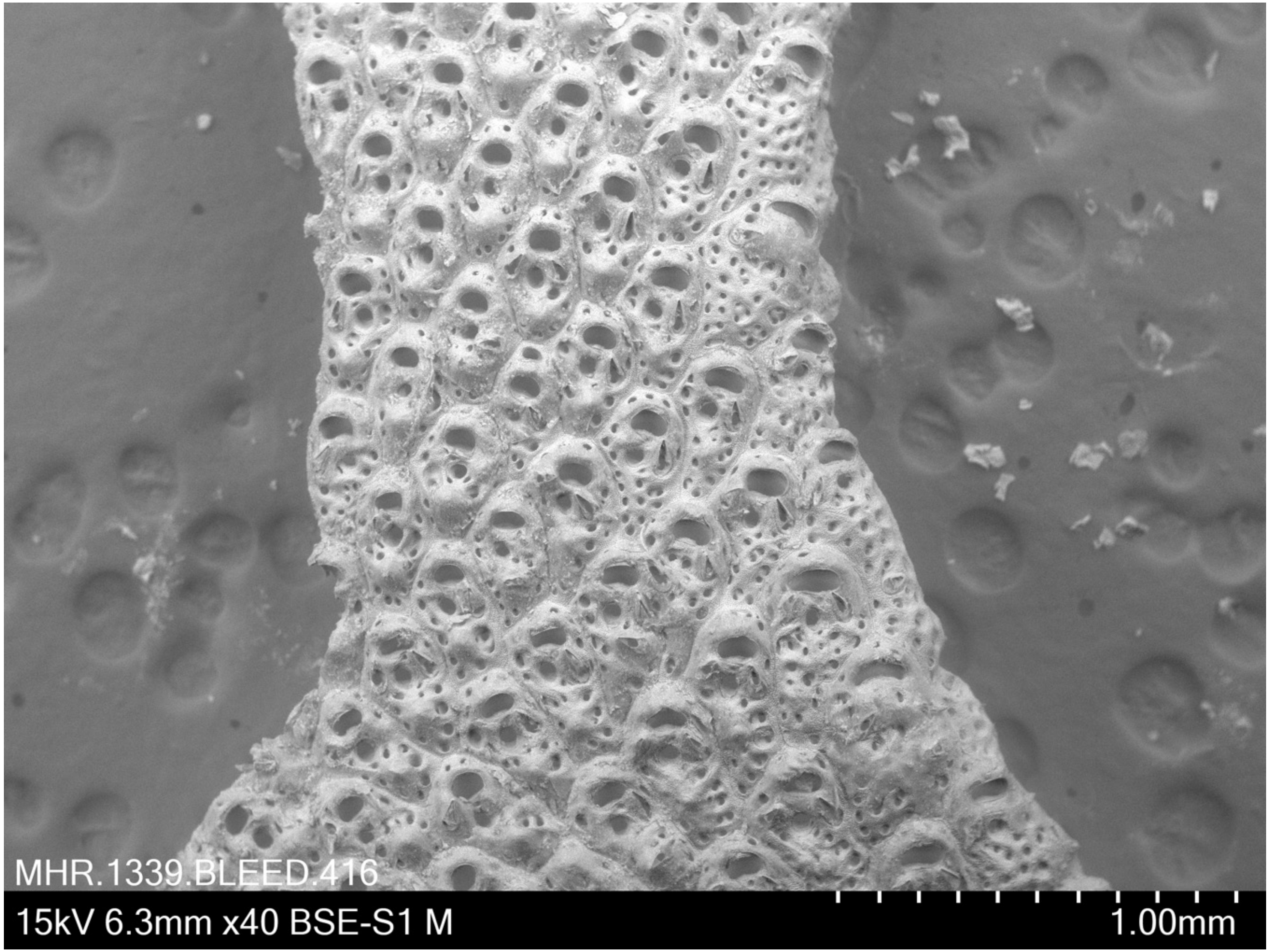

BLEED 417

Adeonella sp. 2

WAM Z90871
NHMO H 1436

Camden Sound
Australia

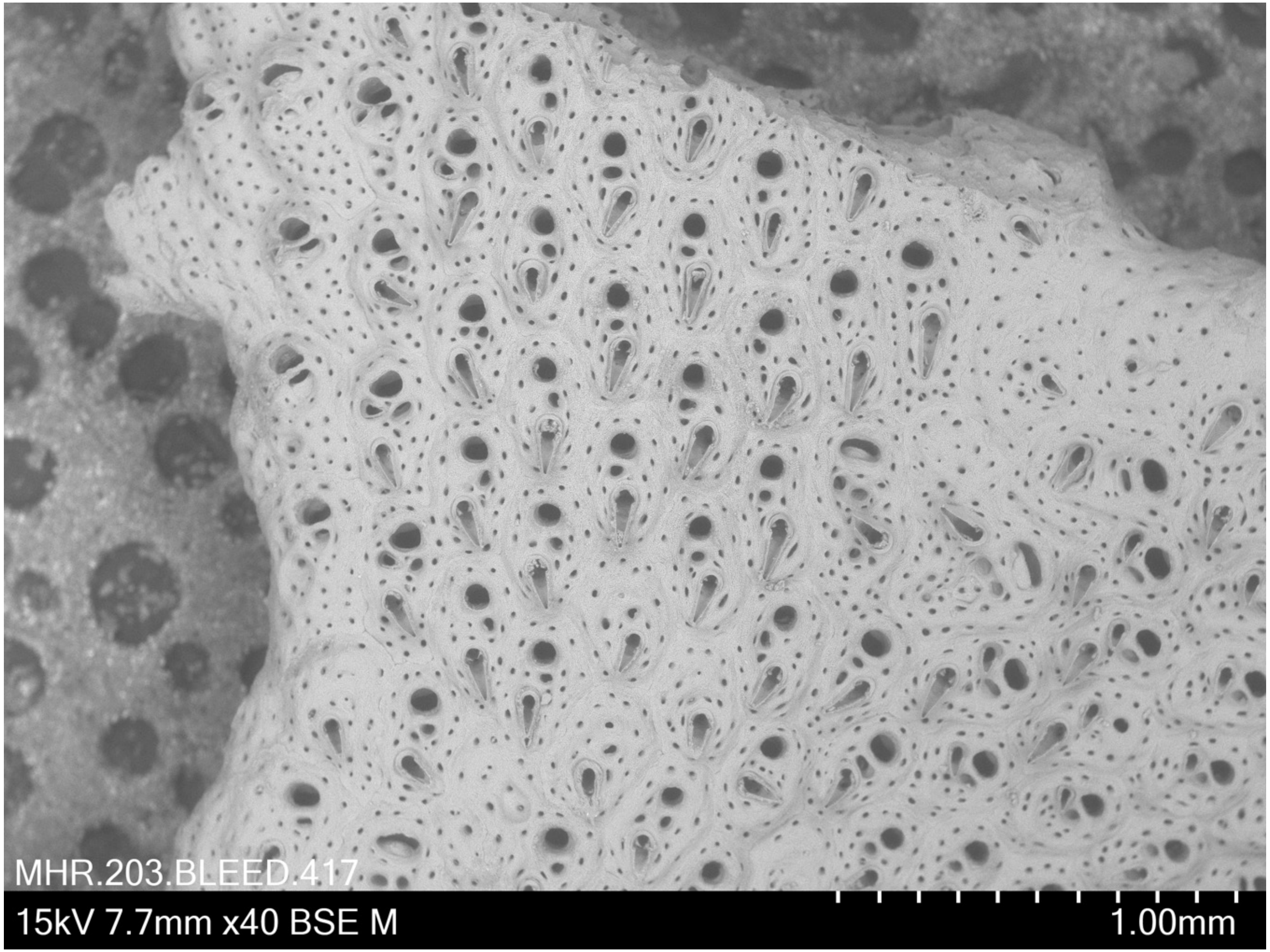

BLEED 393

Adeonella pluscula

NHMO H 1439

Oudekraal, Cape Town
South Africa

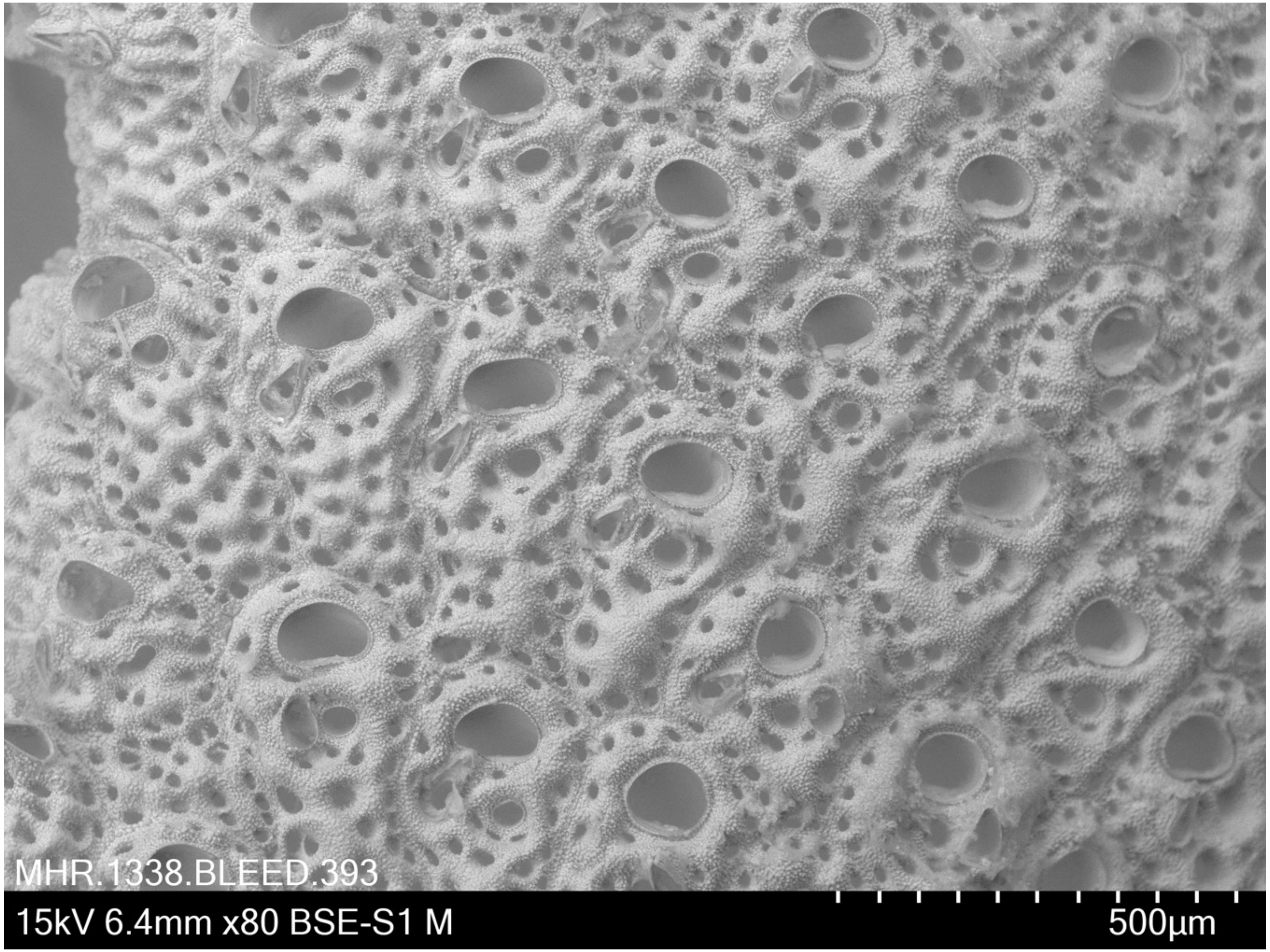

BLEED 410

Adeonella pluscula

NHMO H 1440

Oudekraal, Cape Town
South Africa

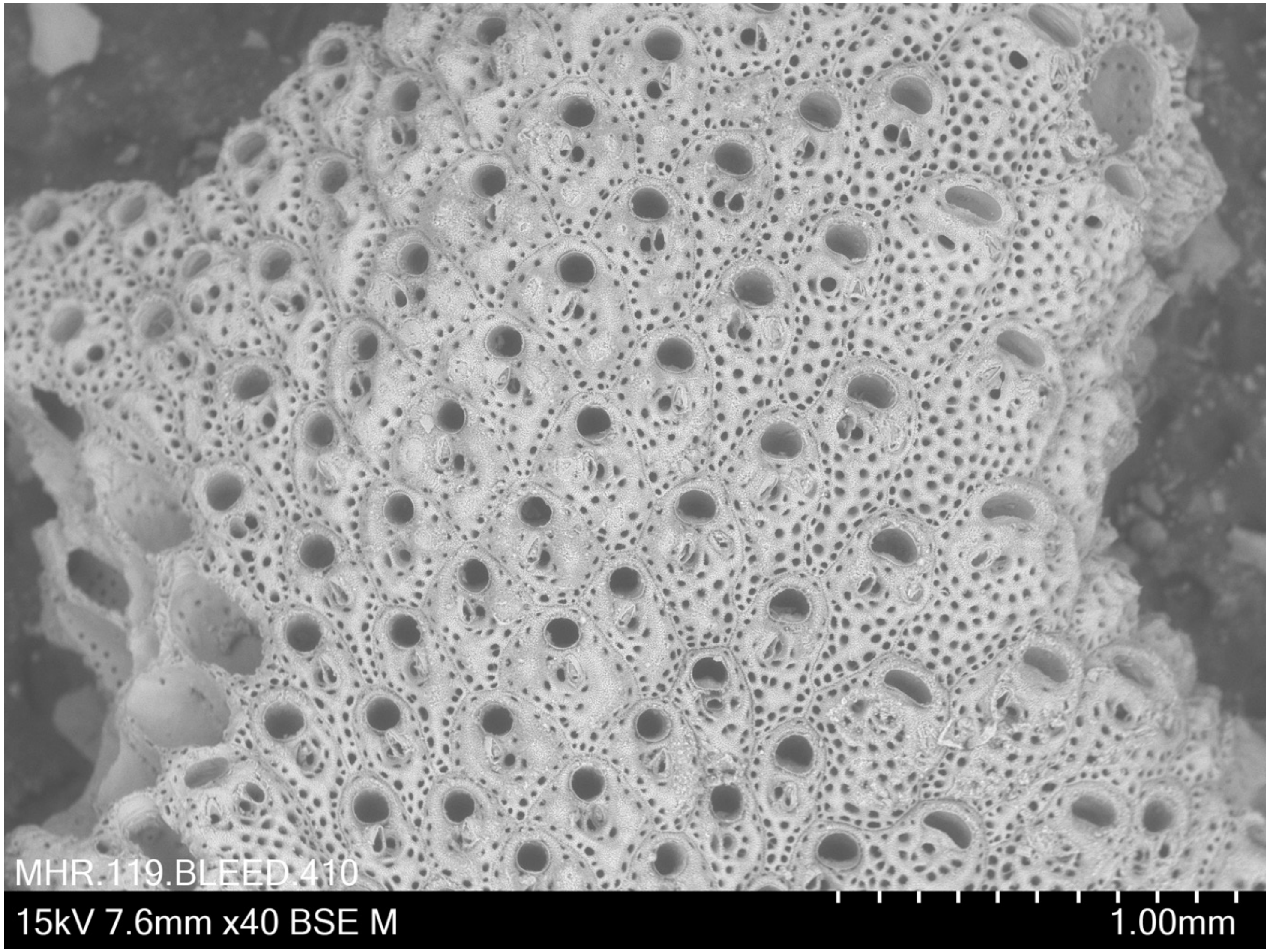

BLEED 391

Laminopora jellyae

NHMO H 1438

Oudekraal, Cape Town
South Africa

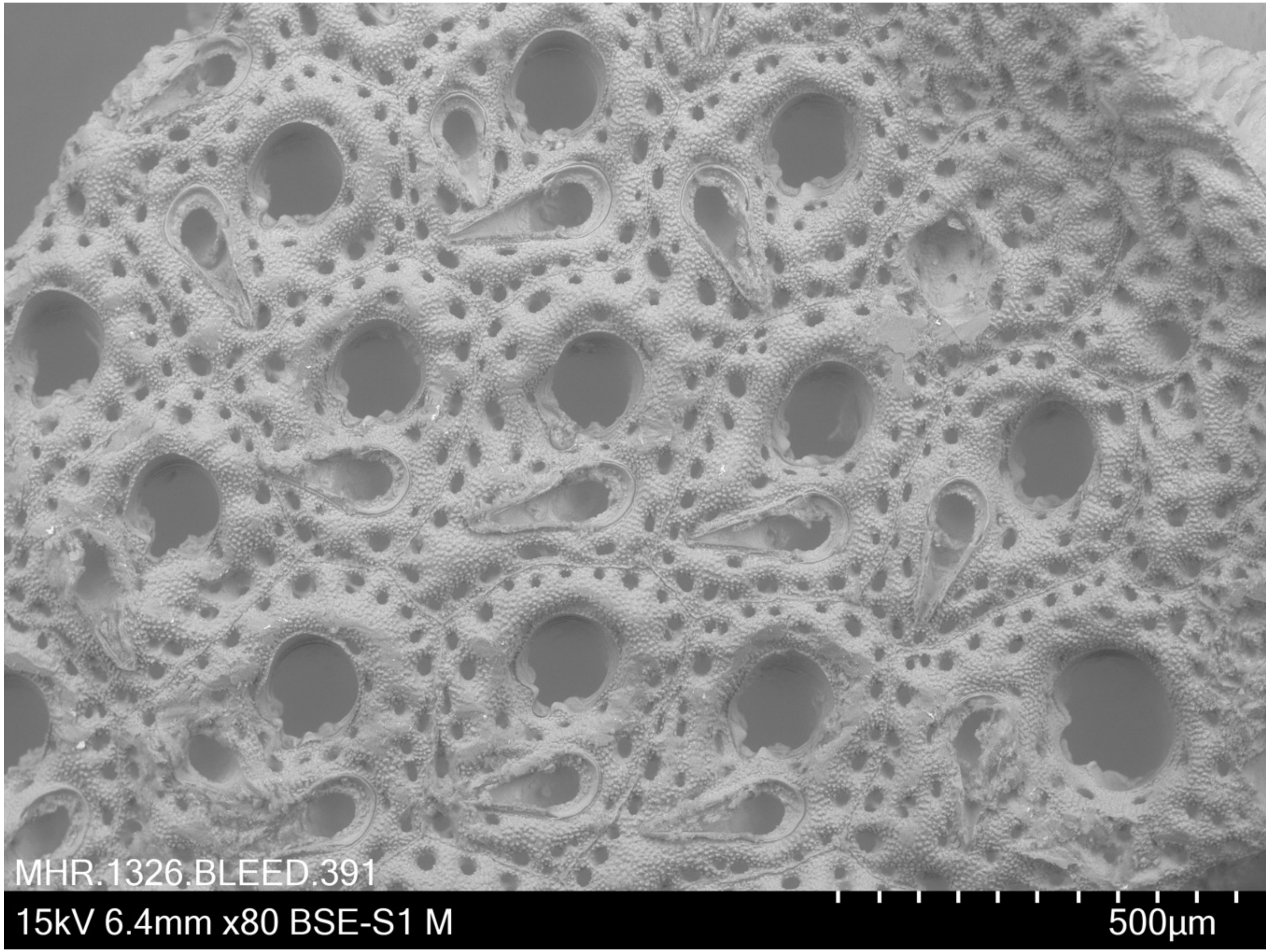

BLEED 408

Laminopora jellyae

NHMO H 1437

Oudekraal, Cape Town
South Africa

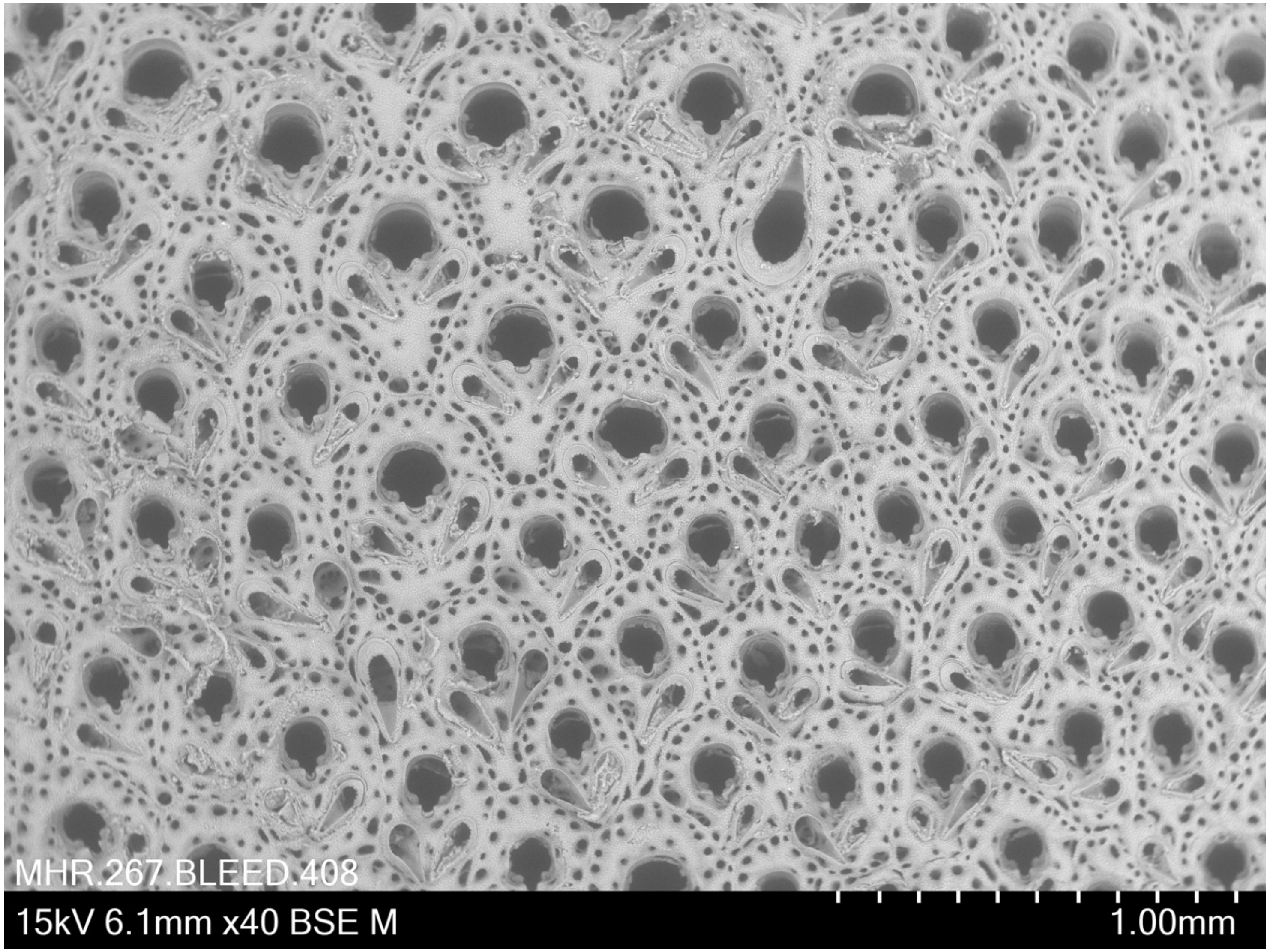

BLEED 373

Laminopora contorta

NHMO H 1441

Cape Verde

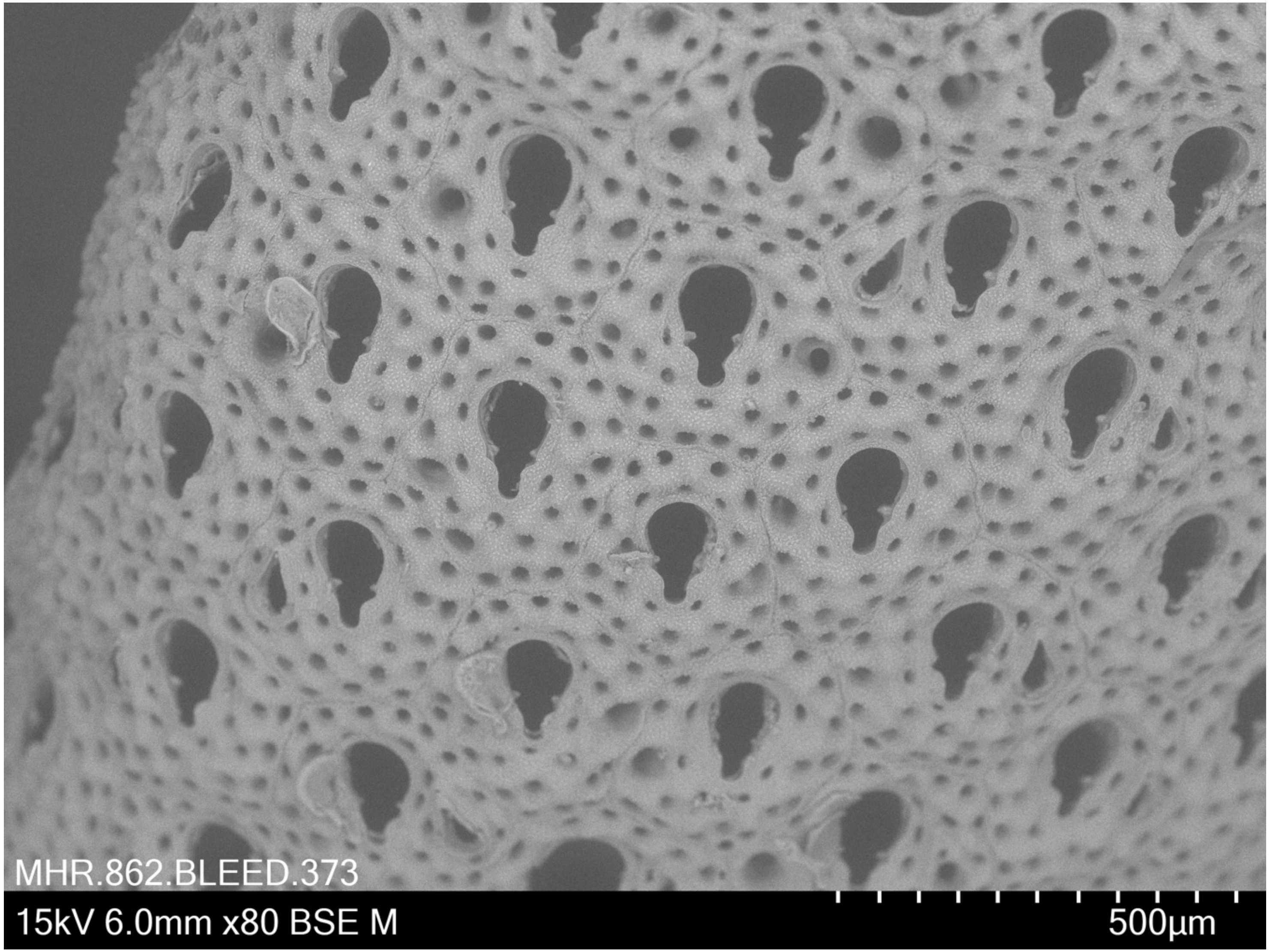

BLEED 401

Reptadeonella bipartita

NHMO H 1429

Ilhas Rasas, Guarapari,
Espirito Santo
Brazil

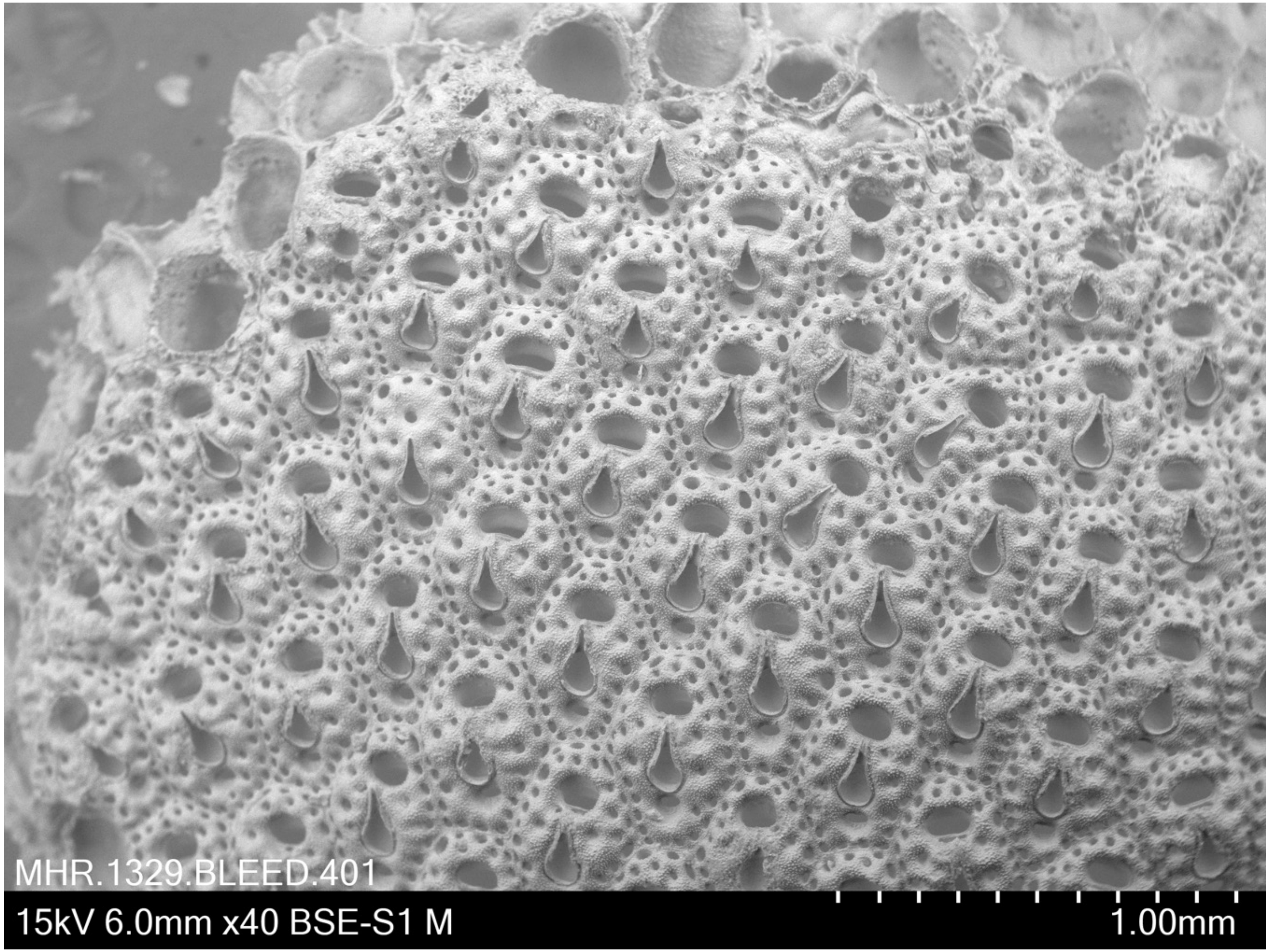

BLEED 396

Reptadeonella brasiliensis

NHMO H 1430

Praia de Pituba,
Salvador, Bahia
Brazil

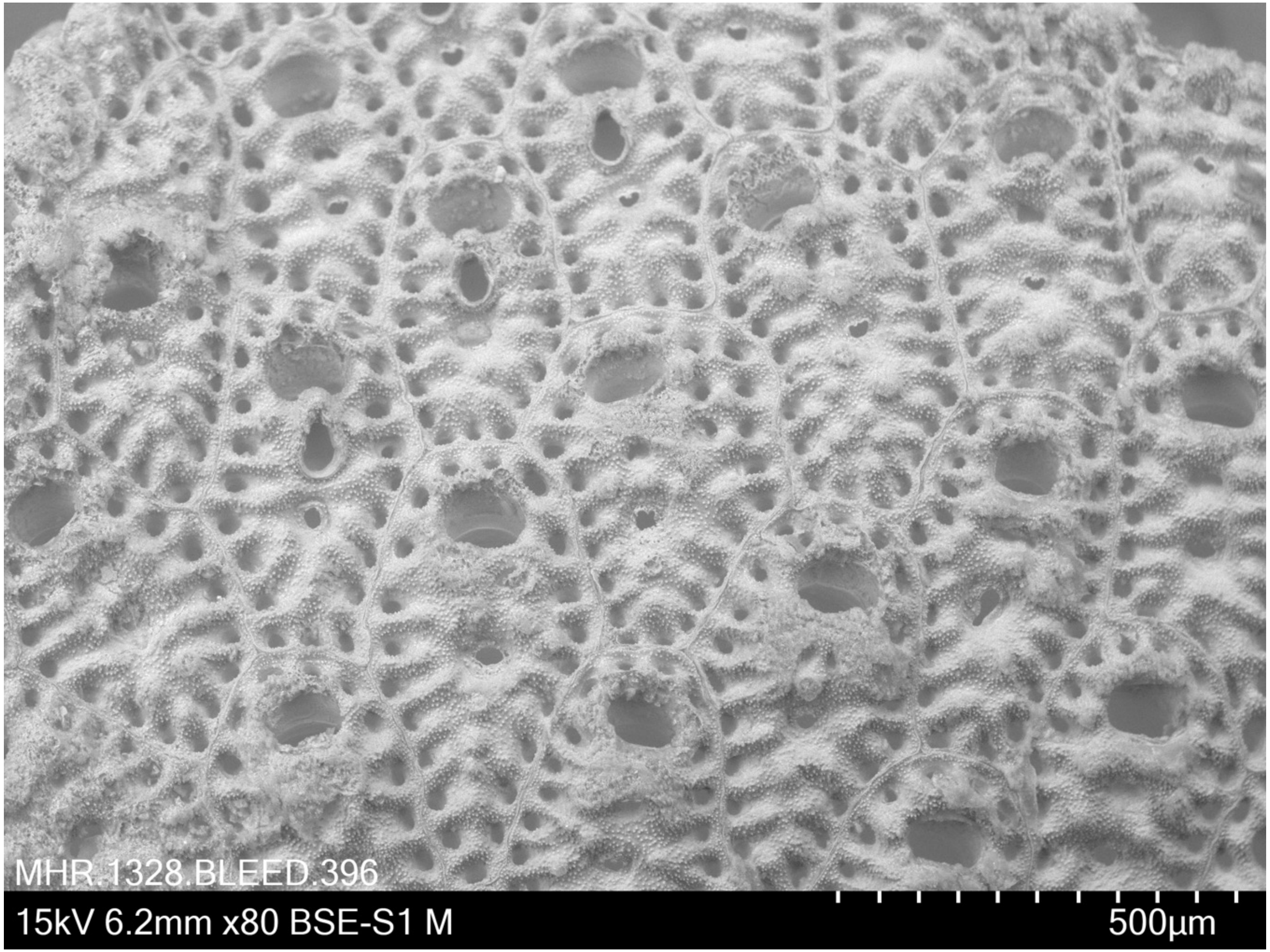

BLEED 41

Reptadeonella aff. *violacea*

NHMO H 1431

Korcula
Croatia

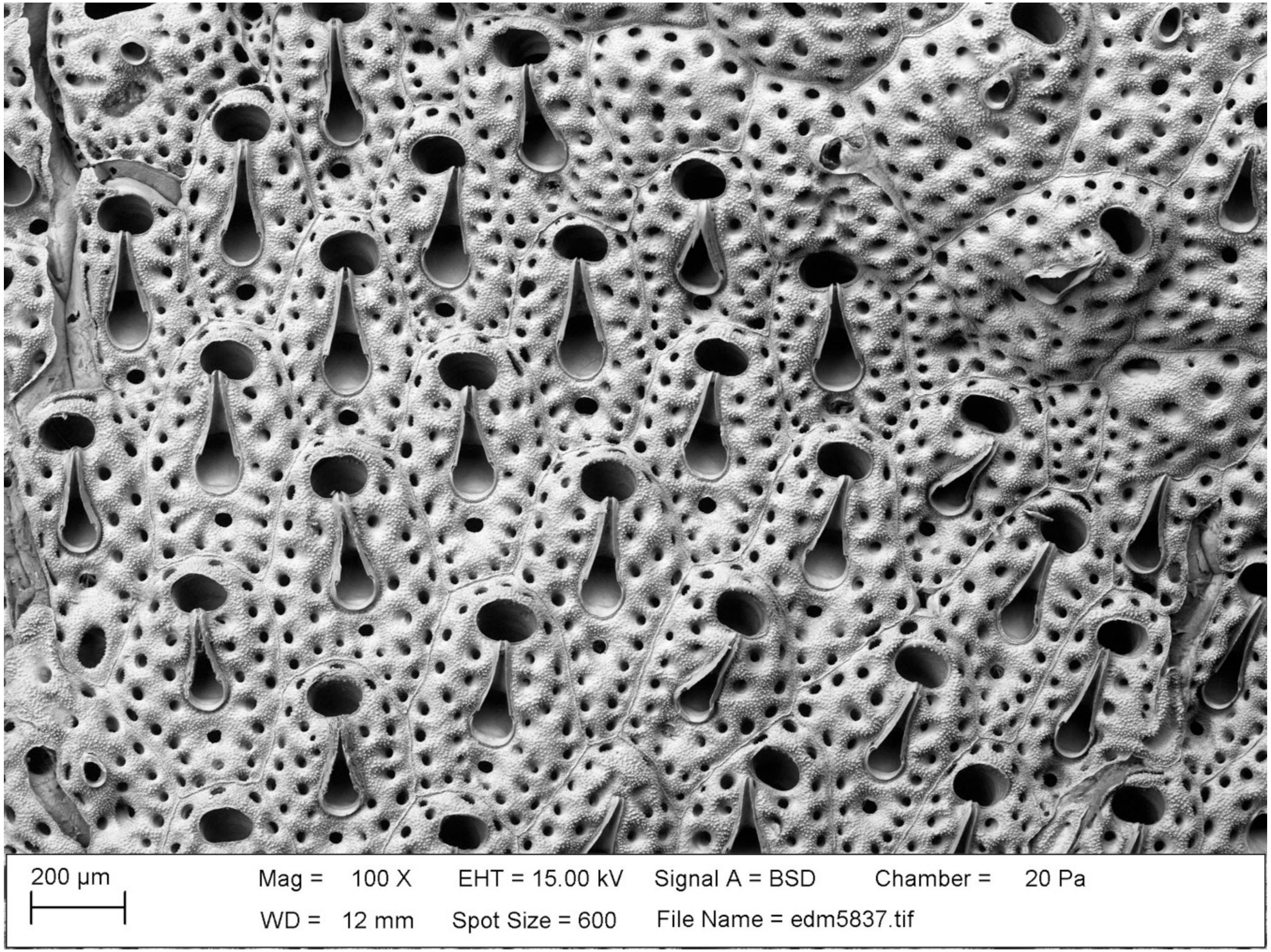

BLEED 302

Cucullipora sp.

NHMO H 1442

Off Bald Island
Australia

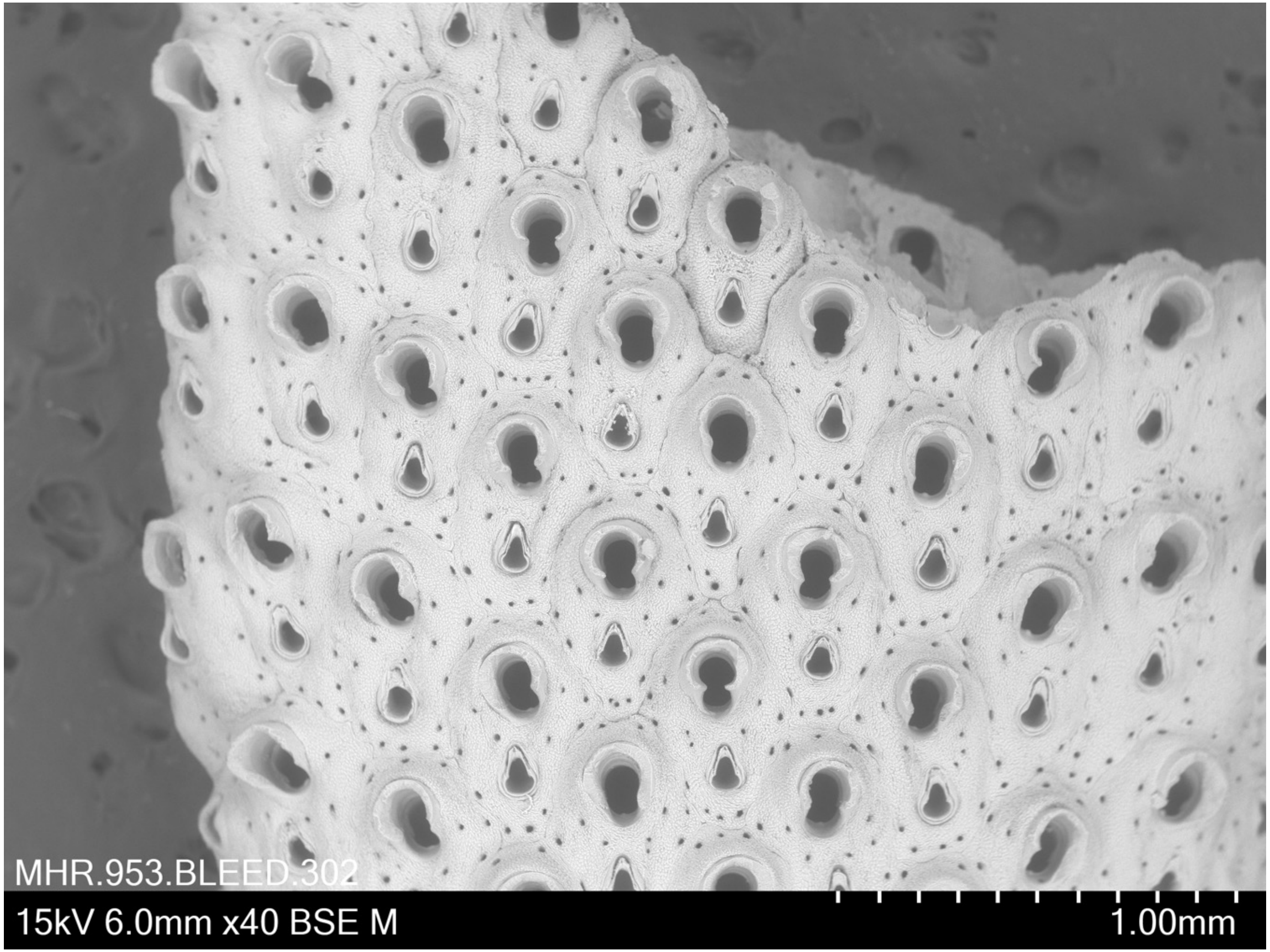

BLEED 38

Adeonella calveti

NHMO H 1445

Oran, Algeria

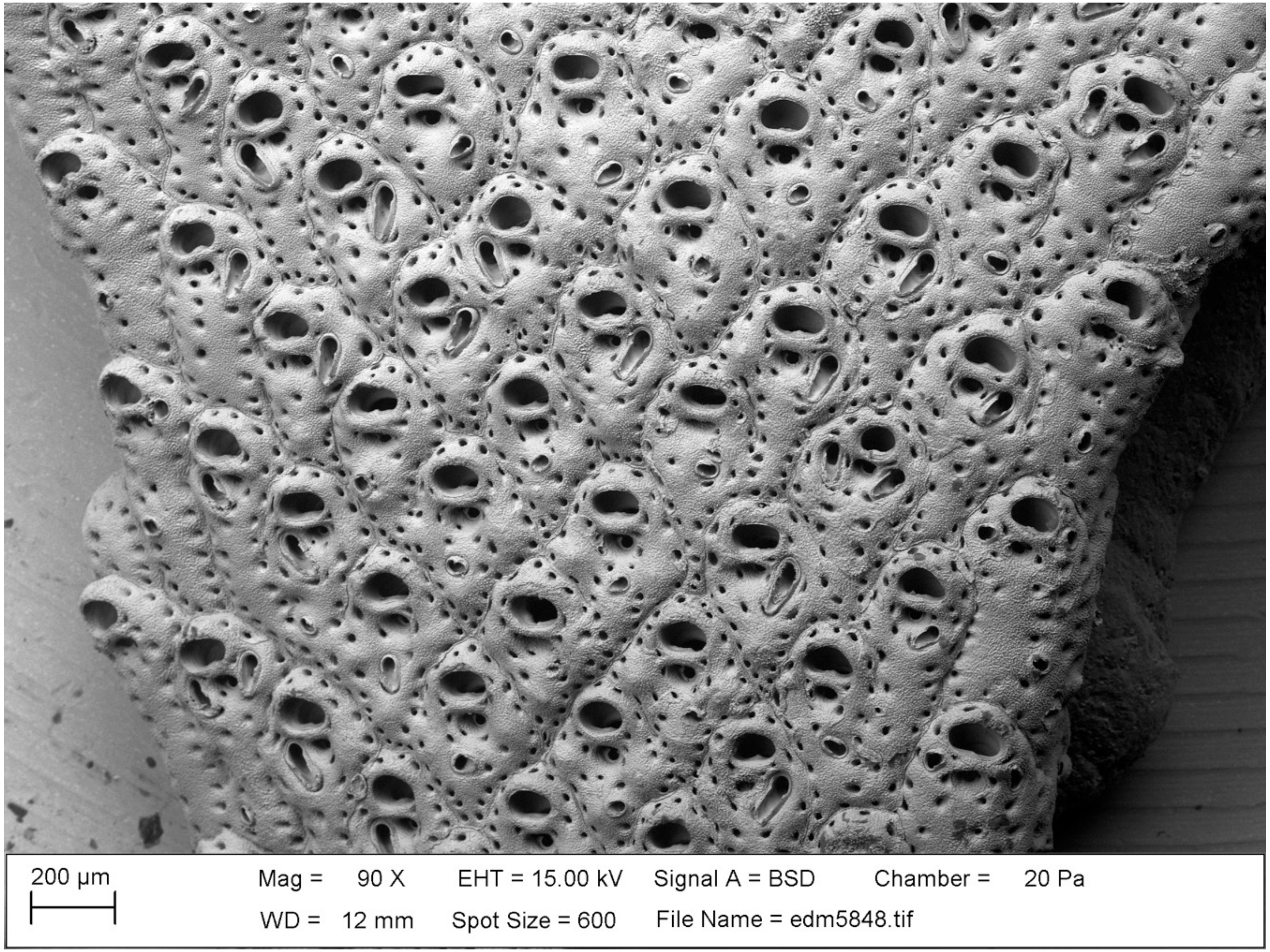

BLEED 39

Adeonella cf. *pallasii*

NHMO H 1444

Cyprus

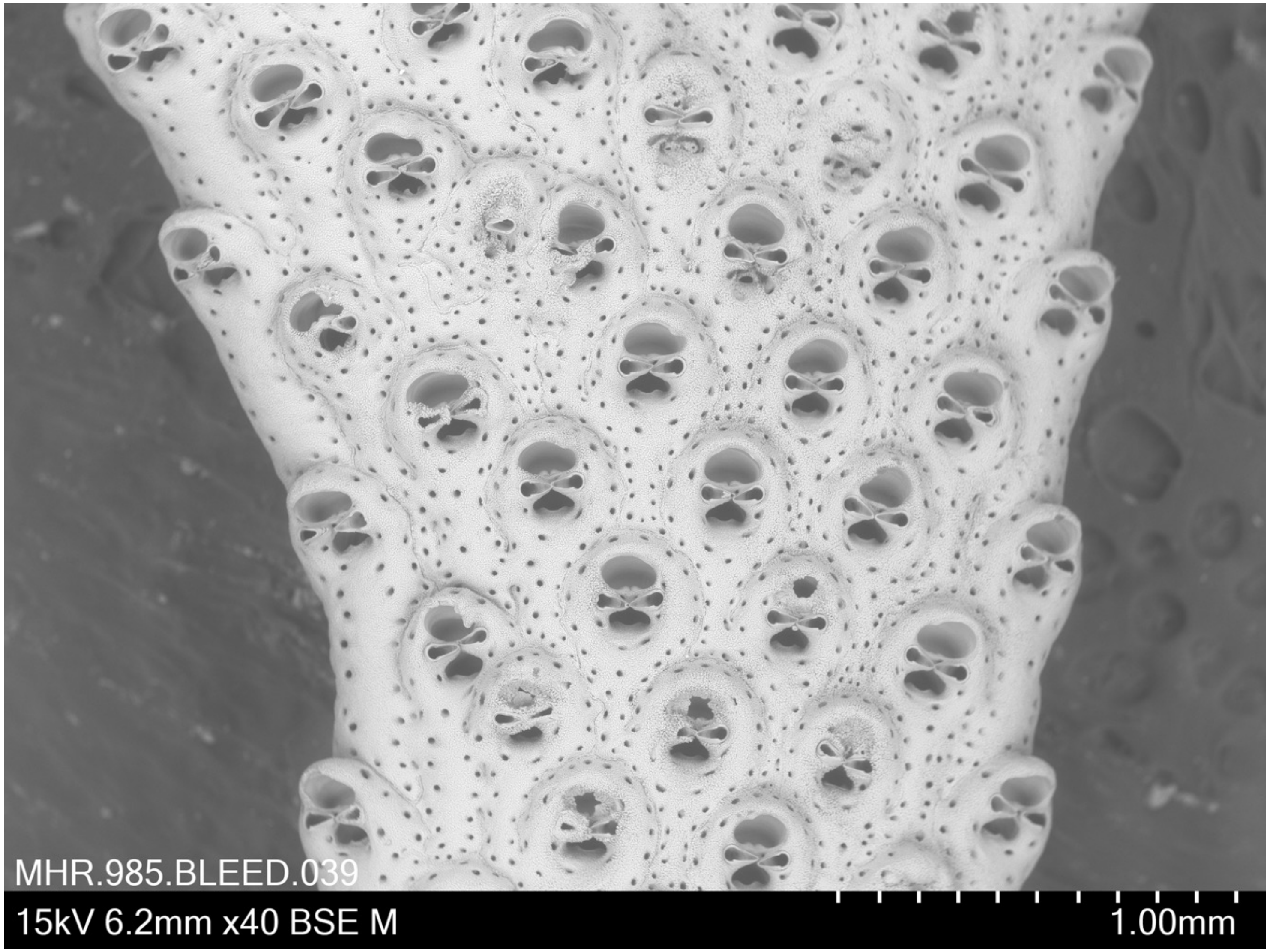

BLEED 40

Adeonella pallasii

NHMO H 1443

Korcula,
Croatia

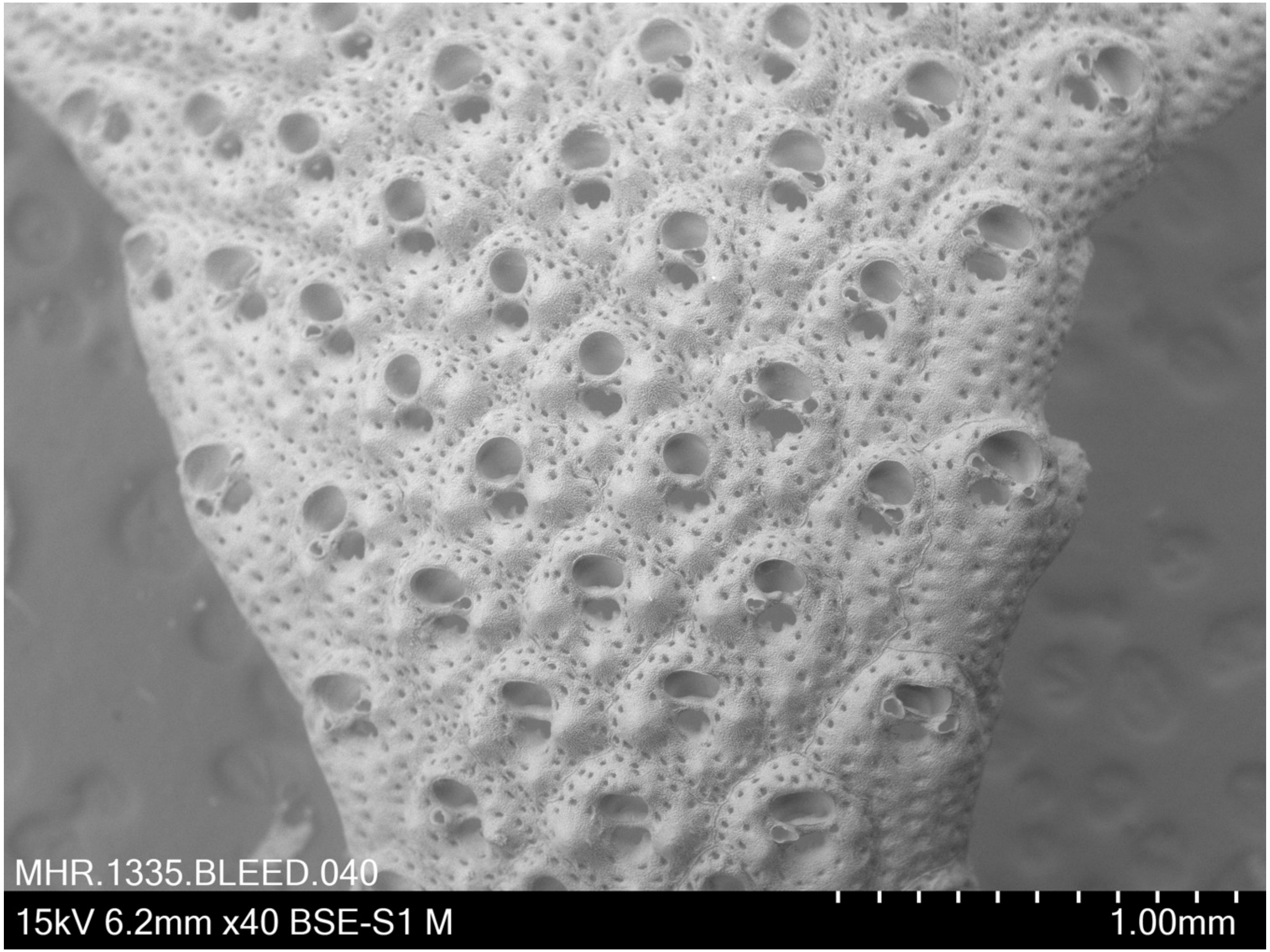

Supplement: Supplementary file 3 — Additional file 3. Scanning Electron Micrographs (SEMs) of dried samples. [file 12862_2019_1563_MOESM3_ESM.pdf]
